# Regularity of non-stationary subdivision: a matrix approach

**DOI:** 10.1007/s00211-016-0809-y

**Published:** 2016-05-12

**Authors:** M. Charina, C. Conti, N. Guglielmi, V. Protasov

**Affiliations:** 1 University of Vienna, Vienna, Austria; 2 DIEF-University of Florence, Florence, Italy; 3 University of L’Aquila and Gran Sasso Science Institute, L’Aquila, Italy; 4 Moscow State University and National Research University Higher School of Economics, Moscow, Russia

**Keywords:** 65D17, 15A60, 39A99

## Abstract

In this paper, we study scalar multivariate non-stationary subdivision schemes with integer dilation matrix *M* and present a unifying, general approach for checking their convergence and for determining their Hölder regularity (latter in the case $M = mI, m \ge 2$). The combination of the concepts of asymptotic similarity and approximate sum rules allows us to link stationary and non-stationary settings and to employ recent advances in methods for exact computation of the joint spectral radius. As an application, we prove a recent conjecture by Dyn et al. on the Hölder regularity of the generalized Daubechies wavelets. We illustrate our results with several examples.

## Introduction

We provide a general, unifying method for convergence and regularity analysis of multivariate non-stationary, i.e. level-dependent, subdivision schemes with an integer dilation matrix *M* whose eigenvalues are all larger than 1 in the absolute value. It has been believed until recently that the joint spectral radius approach, successfully used for the regularity analysis of stationary subdivisions, is not applicable in the non-stationary setting. Our results dismiss this belief. We show that the joint spectral radius techniques are applicable for all non-stationary schemes that satisfy two mild assumptions: all level-dependent masks have the same bounded support and satisfy the so-called *approximate sum rules*. We show that the approximate sum rules are “almost necessary” for convergence and regularity of non-stationary schemes. We derive sharp criteria for convergence of non-stationary schemes in the spaces $C^{\ell }\, , \ \ell \ge 0$, and, in the case $M = mI, m \ge 2$, obtain a formula for the Hölder exponent of their limit functions. The application of our results allows us, e.g. to determine the Hölder regularity of the generalized Daubechies wavelets and, thus, prove the recent conjecture by Dyn et al. [32]. In this paper we focus mainly on subdivision schemes, although our results are applicable to all non-stationary refinable functions, in particular, the ones used for constructions of non-stationary wavelets.

Subdivision schemes are linear iterative algorithms that interpolate or approximate data on a given polygonal mesh. From starting data, such schemes repeatedly compute local, linear weighted averages of sequences of real numbers or point in $\mathbb {R}^s$, $s=2,3$. The weights are real numbers that define the so-called subdivision mask of the scheme. The scheme converges, if the subdivision recursion generates a continuous limit function from every starting data sequence. The first subdivision scheme is the corner cutting algorithm by de Rham [30] that generates smooth curves from the given vertices of a polygon in $\mathbb {R}^2$.

Subdivision schemes are simple to implement, intuitive in use, and possess many other nice properties (linearity, shift-invariance, etc). This motivates their wide popularity in modeling freeform curves and surfaces and in computer animation. The potential of subdivision schemes has recently also become apparent in the context of isogeometric analysis, a modern computational approach that integrates finite element analysis into conventional CAD systems. Thus, in the last ten years, there has been an increase of interest in subdivision schemes. The main questions when analyzing any scheme are its convergence and the regularity of its limit functions. Both of these questions can be answered effectively using the *matrix approach* that reduces these questions to computation of the *joint spectral radius* of a special, compact set of square matrices.

Non-stationary subdivision schemes were introduced to enrich the class of limit functions of stationary schemes and have very different and distinguished properties. Indeed, it is well-known that stationary subdivision schemes are not capable of generating circles, ellipses, or, in general, of generating exponential (quasi-) polynomials $x^\gamma e^{\lambda \cdot x}$, $x \in \mathbb {R}^s$, $\gamma \in \mathbb {N}_0^s$, $\lambda \in \mathbb {C}^s$, while non-stationary schemes generate function spaces that are much richer and include exponential polynomials as well as exponential *B*-splines (see e.g. [2, 27, 35, 46, 48, 53, 63]). This generation property is important in several applications, e.g. in biological imaging [28, 29], geometric design [51, 62, 67] and in isogeometric analysis (see [3, 13, 14, 23] and references therein). The interest in non-stationary subdivision schemes is also due to the fact that they include Hermite schemes that do not only model curves and surfaces, but also their gradient fields. Such schemes are used in geometric modelling and biological imaging, see e.g. [39, 54–56, 65]. Additionally, multi-resolution analysis based on any stationary subdivision scheme uses the same filters at each level of the decomposition and reconstruction. On the contrary, non-stationary wavelet and frame constructions are level adapted and more flexible, see e.g. [32, 37, 48, 50, 66]. Unfortunately, in practice, the use of subdivision is mostly restricted to the class of stationary subdivision schemes. One reason for that is the lack of general methods for their analysis, especially methods for their convergence and regularity analysis. This motivates our study.

The main difficulty is that the matrix approach cannot be directly extended to the non-stationary setting. In the stationary case, the Hölder regularity of subdivision limits is derived from the joint spectral radius of a finite set of linear operators which are restrictions of *transition matrices* of the subdivision scheme to their special linear subspace. In the non-stationary case, one deals with a sequence of transition matrices and a sequence of their corresponding linear subspaces. Both may not converge a priori. Deep analysis of such sequences allows us to prove that the sequence of such linear subspaces does possess a limiting subspace (provided the scheme converges). Moreover, as in the stationary case, we show how to express the Hölder regularity of non-stationary subdivision in terms of the joint spectral radius of the limit points of the sequence of transition matrices restricted to this limiting linear subspace. Both results provide a powerful tool for analysis of non-stationary subdivision schemes. Several numerical examples demonstrate the efficiency of our method.

Finally, note that there is another class of non-stationary schemes that can generate $C^\infty$ limits (Rvachev-type functions) with bounded support, see [33]. The trade-off is that the supports of their level-dependent subdivision masks grow from level to level of the subdivision recursion. Our approach for regularity analysis is based on computations of the joint spectral radius of a compact set of matrices. Therefore, naturally, it does not apply to Rvachev-type schemes, since, the corresponding matrix sets are unbounded. For analysis and applications of Rvachev-type schemes we refer the reader, for example, to [15, 33, 49].

### Framework

Let $M=mI$, $m \ge 2$. Given an initial set of data ${\varvec{c}}^{(1)}:=\{ c^{(1)}(\alpha ) \in \mathbb {R},\ \alpha \in \mathbb {Z}^s\}$ a subdivision scheme iteratively constructs a sequence of progressively denser data by means of *local refinement rules* which are based on the sequence of *subdivision operators* $\{S_{\mathbf {a}^{(k)}},\ k\ge 1\}$. The subdivision operators $S_{\mathbf {a}^{(k)}}: \ell (\mathbb {Z}^s) \rightarrow \ell (\mathbb {Z}^s)$ are linear operators and map coarser sequences ${\varvec{c}}^{(k)} \in \ell (\mathbb {Z}^s)$ into finer sequences ${\varvec{c}}^{(k+1)} \in \ell (\mathbb {Z}^s)$ via the rules

1 $$\begin{aligned} {\varvec{c}}^{(k+1)}:=S_{\mathbf {a}^{(k)}} {\varvec{c}}^{(k)}, \ \ S_{\mathbf {a}^{(k)}} {\varvec{c}}^{(k)}(\alpha ):=\sum _{\beta \in \mathbb {Z}^s} \mathrm{a}^{(k)}(\alpha -M\beta ) c^{(k)}(\beta ),\quad k \ge 1,\quad \alpha \in \mathbb {Z}^s. \end{aligned}$$

The *masks* $\{\mathbf {a}^{(k)},\ k\ge 1\}$ are sequences $\mathbf {a}^{(k)}:=\{\mathrm{a}^{(k)}(\alpha ) \in \mathbb {R}, \ \alpha \in \mathbb {Z}^s\}$ of real numbers and we assume that all $\mathbf {a}^{(k)}$ have bounded supports in $\{0,\ldots ,N\}^s$ with $N \in \mathbb {N}$. To be able to use the joint spectral radius approach, we furthermore assume that the sequence $\{\mathbf {a}^{(k)},\ k\ge 1\}$ is bounded. Such schemes are called either *level-dependent*, or *non-stationary* or *non-homogeneous*. Here we use the term *non-stationary* and denote these type of subdivision schemes by the corresponding collection of subdivision operators $\{S_{\mathbf {a}^{(k)}}, \ k\ge 1 \}$. A subdivision scheme whose refinement rules are level independent is said to be *stationary* (see [5], for example) and, for all $k \ge 1$, is defined by the same sequence $\mathbf {a}:=\{\mathrm{a}(\alpha ) \in \mathbb {R},\ \alpha \in \mathbb {Z}^s\}$ of refinement coefficients, i.e. $\mathbf {a}^{(k)}=\mathbf {a}$, $k \ge 1$. The corresponding subdivision scheme is therefore denoted by $S_\mathbf {a}$. There is a multitude of results on convergence and regularity of stationary subdivision schemes in the literature (for example see [5, 11, 12, 43, 47, 57] and references therein). These results rely on polynomial generation and reproduction properties of subdivision operators and employ the so-called restricted spectral radius or the joint spectral radius techniques. It has been believed until recently that these two concepts have no immediate application in the non-stationary setting. The reason for this belief is that convergent non-stationary schemes do not necessarily generate or reproduce any polynomial spaces, see e.g. [19].

In this paper, we make use of the concepts of *approximate sum rules* and *asymptotic similarity* to link stationary and non-stationary settings and show how to employ the *joint spectral radius* for smoothness analysis of non-stationary schemes. This allows us to provide a general and unifying approach for the analysis of convergence and regularity of a vast majority of non-stationary subdivision schemes. Our results generalize the existing well-known methods in [18, 34, 36], which only allow us to check convergence and Hölder regularity of special instances of non-stationary schemes. In fact, the sufficient conditions in [34] are based on the concept of *asymptotic equivalence* which we recall in the following Definition 1, where *E* is a set of representatives of $\mathbb {Z}^s/M \mathbb {Z}^s$, i.e. $E \simeq \{0,\ldots ,m-1\}^s$.

#### Definition 1

Let $\ell \ge 0$. Two non-stationary schemes $\{S_{\mathbf {a}^{(k)}}, \ k \ge 1\}$ and $\{S_{\mathbf {b}^{(k)}}, \ k \ge 1\}$ are called *asymptotically equivalent (of order* $\ell$), if they satisfy

2 $$\begin{aligned} \sum _{k=1}^\infty m^{k \ell } \Vert S_{\mathbf {a}^{(k)}}-S_{\mathbf {b}^{(k)}}\Vert _\infty <\infty ,\quad \hbox {for} \ \Vert S_{\mathbf {a}^{(k)}}\Vert _\infty :=\max _{\varepsilon \in E}\left\{ \sum _{\alpha \in \mathbb {Z}^s}|\mathrm{a}^{(k)}(M\alpha +\varepsilon )|\right\} .\quad \end{aligned}$$

In the case of $M=2I$ and under certain additional assumptions on the schemes $\{S_{\mathbf {a}^{(k)}}, \ k \ge 1\}$ and $\{S_{\mathbf {b}^{(k)}}, \ k \ge 1\}$, the method in [34] allows us to determine the regularity of $\{S_{\mathbf {a}^{(k)}}, \ k \ge 1\}$ from the known regularity of the asymptotically equivalent scheme $\{S_{\mathbf {b}^{(k)}}, \ k \ge 1\}$. In [36], in the univariate binary case, the authors relax the condition of asymptotic equivalence. They require that the $D^j$th derivatives of the symbols

$$\begin{aligned} a_*^{(k)}(z):=\sum _{\alpha \in \mathbb {Z}} \mathrm{a}^{(k)}(\alpha )z^\alpha , \quad z \in \mathbb {C}{\setminus } \{0\}, \quad k\ge 1, \end{aligned}$$

of the non-stationary scheme $\{S_{\mathbf {a}^{(k)}}, \ k\ge 1 \}$ satisfy

3 $$\begin{aligned} |D^j a_*^{(k)}(-1)| \le C \, 2^{-(\ell +1-j)k}, \quad j=0,\ldots ,\ell , \quad \ell \ge 0,\quad C \ge 0, \end{aligned}$$

and, additionally, assume that the non-stationary scheme is asymptotically equivalent (of order 0) to some stationary scheme. The conditions in (3) can be seen as a generalization of the so-called sum rules in (4). In the stationary case, sum rules are necessary for smoothness of subdivision, see e.g [4, 5, 42, 45].

#### Definition 2

Let $\ell \ge 0$. The symbol

$$\begin{aligned} a_*(z)=\sum _{\alpha \in \mathbb {Z}^s} \mathrm{a}^{(k)}(\alpha )z^\alpha , \quad z \in \mathbb {C}^s {\setminus } \{0\}, \end{aligned}$$

satisfies sum rules of order $\ell +1$ if

4 $$\begin{aligned} {a}_*(1)=m^s \quad \hbox {and}\quad \max _{|\eta | \le \ell }\ \max _{\epsilon \in \Xi {\setminus } \{1\}} | D^\eta a_*(\epsilon )|=0. \end{aligned}$$

In the above definition, $\Xi :=\{e^{-i\frac{2\pi }{m}\varepsilon }, \ \varepsilon \in E\}$ and $D^\eta$, $\eta \in \mathbb {N}_0^s$, denotes the $\eta$th directional derivative.

In the spirit of (3), in this paper, we present a generalization of the notion of sum rules which we call *approximate sum rules*.

#### Definition 3

Let $\ell \ge 0$. The sequence of symbols $\{a^{(k)}_*(z),\ \ k \ge 1\}$ satisfies approximate sum rules of order $\ell +1$, if

5 $$\begin{aligned} \mu _k:=|{a}_*^{(k)}(1)-m^s| \quad \hbox {and}\quad \delta _k\ :=\ \max _{|\eta | \le \ell } \max _{\epsilon \in \Xi {\setminus } \{1\}} |{m^{-k|\eta |} D^\eta {a}_*^{(k)}(\epsilon )|} \end{aligned}$$

satisfy

6 $$\begin{aligned} \sum _{k=1}^{\infty } \mu _k< \infty \quad \hbox {and}\quad \sum _{k=1}^{\infty } m^{\, k\ell }\, \delta _k < \infty . \end{aligned}$$

We call the sequence $\{\delta _k, \ k \ge 1\}$ *sum rule defects*. If the sequences $\{\mu _k, \ k \ge 1\}$ and $\{\delta _k, \ k \ge 1\}$ are zero sequences, then the symbols of the corresponding non-stationary scheme satisfy sum rules of order $\ell +1$.

Note that, even in the univariate binary case, the assumption on $\{\delta _k, \ k \ge 1\}$ in (6), i.e.

7 $$\begin{aligned} \sum _{k=1}^\infty 2^{\ell k} \delta _k < \infty , \quad \delta _k:=\ \max _{j \le \ell } {2^{-k\, j} | D^j {a}_*^{(k)}(-1)|}, \end{aligned}$$

is less restrictive than the decay condition on $\{\delta _k, \ k \ge 1\}$ in (3). In Theorem 1, we showed that approximate sum rules are close to being necessary conditions for regularity of non-stationary schemes, i.e. even in the univariate binary setting, the sum rules defects $\{\delta _k, \ k\ge 1\}$ must decay faster than $2^{\, -\ell k}$, if the limit functions of the scheme are $C^\ell$. Indeed, in the binary univariate case, we show that under assumption of asymptotical similarity (see Definition 5) to a stationary scheme whose basic refinable function is stable, the $C^\ell$-regularity of the non-stationary scheme implies that the sum rules defects $\{\delta _k, \ k\ge 1\}$ must decay faster than $2^{\, -\ell k}$. Clearly, there is still a gap between the corresponding necessary condition $\lim _{k \rightarrow \infty } 2^{-\ell k}\delta _k = 0$ and one of the sufficient conditions $\sum _{k \in \mathbb {N}} 2^{-\ell k} \delta _k < \infty$. See also Example 1. Moreover, in [22], the authors proved that this decay rate of the sum rules defects is necessary for generation of $\ell$ linearly independent functions from $\{x^\gamma e^{\lambda x}, \ \gamma \in \mathbb {N}_0, \ \lambda \in \mathbb {C}\}$. This resembles the stationary setting and motivates our multivariate convergence and smoothness analysis of non-stationary schemes.

In [18], in the univariate binary non-stationary setting, milder sufficient conditions than asymptotic equivalence are essentially derived under the assumptions that the scheme $\{S_{\mathbf {a}^{(k)}},\ \ k \ge 1\}$ is *asymptotically similar* to a suitable non-stationary scheme $\{S_{\mathbf {b}^{(k)}},\ \ k \ge 1\}$, i.e. $\lim \nolimits _{k\rightarrow \infty }\Vert \mathbf {a}^{(k)}-\mathbf {b}^{(k)}\Vert _\infty =0\,$, and both satisfy sum rules of order 1. Here we generalize the notion of asymptotic similarity making use of the following concept of *set of limit points* of a sequence of masks.

#### Definition 4

For the mask sequence $\{\mathbf {a}^{(k)},\ \ k \ge 1\}$ we denote by ${\mathcal A}$ the set of its limit points, i.e. the set of masks $\mathbf {a}$ such that

$$\begin{aligned} \mathbf {a}\in {\mathcal A},\quad \hbox {if} \quad \exists \{k_n,\ n\in \mathbb {N}\}\quad \text{ such } \text{ that }\quad \lim _{n\rightarrow \infty }\mathbf {a}^{(k_n)}=\mathbf {a}. \end{aligned}$$

The following definition of *asymptotic similarity* generalizes the one given in [18]. This notion allows us to relate the properties of non-stationary subdivision schemes to the corresponding properties of the stationary masks in $\mathcal A$.

#### Definition 5

Two non-stationary schemes $\{S_{\mathbf {a}^{(k)}},\ \ k \ge 1\}$ and $\{S_{\mathbf {b}^{(k)}},\ \ k \ge 1\}$ are called *asymptotically similar*, if their sets of limit points coincide.

### Summary of the results

For the reader’s convenience, we summarize here the main results presented in this paper. The details are given in Sect. 3.

In the rest of the paper we assume that the symbols $\{{a}_*^{(k)}(z), \ k \ge 1\}$ satisfy approximate sum rules and are re-scaled in such a way that ${a}_*^{(k)}(1)=m^s$, $k \ge 1$. In this case $\mu _k$ in (5) are equal to zero for all $k \ge 1$ and do not affect our convergence and regularity analysis. On the contrary, if the sequence $\{\mu _k, \ k \ge 1\}$ is not summable, then such a re-scaling can change the properties of the scheme, see Example 1.

One of our results states that even in the univariate case approximate sum rules are close to being necessary for convergence and smoothness of non-stationary subdivision schemes.

#### Theorem 1

Let $\ell \ge 0$. Assume that a univariate binary subdivision scheme $S_\mathbf {a}$ is convergent and its basic refinable limit function is stable. Assume, furthermore, that $\mathbf {a}=\lim _{k \rightarrow \infty } \mathbf {a}^{(k)}$ and the non-stationary subdivision scheme $\{S_\mathbf {a}^{(k)},\ k\ge 1\}$ converges to $C^\ell$ limit functions. Then $\lim _{k \rightarrow \infty } 2^{\, \ell k} \delta _k = 0$ for $\{\delta _k, \ k \ge 1\}$ in (7).

The proof of Theorem 1 is given in Sect. 4. Thus, if the scheme converges to a $C^{\ell }$ limit function, then the sum rule defects $\{\delta _k, \ k \ge 1\}$ decay faster than $2^{\, - \ell k}$, i.e., satisfy $\delta _k \, = \, o(2^{\, - \ell k})$. This does not imply the approximate sum rules, i.e. $\sum _{k \in \mathbb {N}}\delta _k 2^{\, \ell k} < \infty$, but is close to this condition. Thus, Theorem 1 indicates that approximate sum rules is a natural assumption for convergence to a $C^{\ell }$ limit, and cannot be relaxed by much.

In the stationary case, the Hölder regularity of the subdivision limits, as well as the rate of convergence of the corresponding subdivision scheme $S_{\mathbf {a}}$, are determined explicitly in terms of the joint spectral radius of the set of certain square matrices which are derived from the subdivision mask $\mathbf {a}$ and depend on the order of sum rules satisfied by $a_*(z)$. Since, in the non-stationary setting, one cannot assume that all subdivision symbols $\{a^{(k)}_*(z),\ k\ge 1\}$ satisfy sum rules, see [10, 20], the concept of the joint spectral radius is not directly applicable and has no straightforward generalization. For this reason, in Theorem 2, we establish a link between stationary and non-stationary settings via the set ${\mathcal A}$ of limit points of $\{\mathbf {a}^{(k)},\ k\ge 1\}$ and provide sufficient conditions for $C^\ell$-convergence, $\ell \ge 0$, and Hölder regularity of non-stationary schemes. Under $C^\ell$-convergence we understand the convergence of subdivision in the norm of $C^\ell (\mathbb {R}^s)$, see Definition 9 in Sect. 2. Note that $C^0$-convergence is the usual convergence of subdivision in $\ell _\infty$ norm and $C^\ell$-convergence implies the convergence of the scheme to $C^\ell$ limit functions, but not vice versa, see Definition 6 in Sect. 2. As in the stationary setting, each mask in the limit set ${\mathcal A}$ determines a set of transition matrices, see e.g. (13). We denote the collection of the restrictions of all these transition matrices to a given finite dimensional difference subspace $V_\ell$ by ${\mathcal T}_{{\mathcal A}}|_{V_\ell }$, see Sect. 2 for more details. Theorem 2 states that $C^\ell$-convergence and Hölder regularity of non-stationary schemes is determined by the joint spectral radius $\rho _{\mathcal A}$ of this collection ${\mathcal T}_{{\mathcal A}}|_{V_\ell }$.

#### Theorem 2

Let $\ell \ge 0$ and $\{\delta _k, \ k\ge 1\}$ be defined in (5). Assume that the symbols of $\{ S_{{\mathbf a}^{(k)}}, \ k \ge 1\}$ satisfy approximate sum rules of order $\ell +1$ and $\rho _{\mathcal A}:=\rho \left( {\mathcal T}_{{\mathcal A}}|_{V_\ell }\right) <m^{-\ell }$, where ${\mathcal A}$ is the set of limit points of $\{\mathbf {a}^{(k)},\ k\ge 1\}$. Then the non-stationary scheme $\{ S_{{\mathbf a}^{(k)}}, \ k \ge 1\}$ is $C^{\ell }$-convergent and the Hölder exponent $\alpha$ of its limit functions satisfies

$$\begin{aligned} \alpha \ \ge \ \min \, \Bigl \{-\log _m \rho _{\mathcal A}\, , \, - \limsup \limits _{k \rightarrow \infty }\frac{\log _m \delta _k}{k}\Bigr \}. \end{aligned}$$

The proof of Theorem 2 is given in Sect. 3.4. Thus, in the non-stationary case, the smoothness of the limit function depends not only on the joint spectral radius of the matrices in $\mathcal A$, but also on the rate of decay of the sum rules defects $\{\delta _k, \ k \ge 1\}$.

Note that, as in the stationary case, the order of approximate sum rules satisfied by the symbols of a non-stationary scheme can be much higher than its regularity.

For applications of Theorem 2 to parameter dependent non-stationary schemes see [8]. There are several immediate important consequences of Theorem 2 that generalize the corresponding results in [18, 34, 36]. For example the following Corollary extends the results in [18] with respect to the dimension of the space, the regularity of the limit functions and the more general notion of asymptotic similarity given in Definition 5.

#### Corollary 1

Let $\ell \ge 0$. Assume that the symbols of the scheme $\{ S_{{\mathbf a}^{(k)}}, \ k \ge 1\}$ satisfy sum rules of order $\ell +1$ and $\rho _{\mathcal A}:=\rho \left( {\mathcal T}_{{\mathcal A}}|_{V_\ell }\right) <m^{-\ell }$, where ${\mathcal A}$ is the set of limit points of $\{ {\mathbf a}^{(k)}, \ k \ge 1\}$. Then any other asymptotically similar scheme $\{S_{{\mathbf b}^{(k)}}, \ k \ge 1\}$ whose symbols satisfy sum rules of order $\ell +1$ is $C^\ell$-convergent and the Hölder exponent of its limit functions is $\alpha \ \ge -\log _m \rho _{\mathcal A}$.

Theorem 2 provides a lower bound for the Hölder exponent of the subdivision limits, whereas the next result allows us to determine its exact value, under slightly more restrictive assumptions.

#### Theorem 3

Let $\ell \ge 0$. Assume that a stationary scheme $S_{\mathbf {a}}$ is $C^\ell$-convergent with the stable refinable basic limit function $\phi$ whose Hölder exponent $\alpha _\phi$ satisfies $\ell \le \alpha _\phi <\ell +1$. If the symbols of the scheme $\{ S_{{\mathbf a}^{(k)}}, \ k \ge 1\}$ satisfy approximate sum rules of order $\ell +1$, $\lim _{k \rightarrow \infty } \mathbf {a}^{(k)}=\mathbf {a}$ and, additionally

$$\begin{aligned} \limsup _{k \rightarrow \infty } \delta _k^{1/k} < \rho _\mathbf {a}:= \rho (\{T_{\varepsilon , \mathbf {a}}|_{V_\ell }, \ \varepsilon \in E\}), \end{aligned}$$

then the scheme $\{ S_{{\mathbf a}^{(k)}}, \ k \ge 1\}$ is $C^\ell$-convergent and the Hölder exponent of its limit functions is also $\alpha _\phi$.

The proof of Theorem 3 is given in Sect. 3.5. An important special class of non-stationary schemes that satisfy assumptions of Theorem 3 are the schemes whose symbols satisfy sum rules of order $\ell +1$, see Corollary 4 in Sect. 3.5.

The main application of Theorem 3 is the proof of the following conjecture by Dyn et al. stated in [32].

#### Conjecture 1

([32]) The Hölder regularity of every generalized Daubechies type wavelet is equal to the Hölder regularity of the corresponding classical Daubechies wavelet.

We prove this conjecture in Theorem 5 and compute some of the corresponding Hölder exponents, see Sect. 3.6.1.

This paper is organized as follows. In Sect. 2, we summarize important known fact about stationary and non-stationary subdivision schemes. The proofs of the results stated in Sect. 1.2 are given in Sect. 3. In particular, in Sect. 3.3, we provide sufficient conditions for convergence of non-stationary subdivision schemes whose symbols satisfy assumptions of Theorem 2 with $\ell =0$. The argument in the proof of the corresponding Theorem 4 is actually independent of the choice of the dilation matrix *M*. For that reason we give a separate proof of convergence and, then, in Sect. 3.4, present the proof of the more general statement of Theorem 2. In Sect. 3.5, we give the proof of Theorem 3. We illustrate our convergence and regularity results with several deliberately simple examples in Sect. 3.6. There we also prove Conjecture 1 formulated in [32] about the regularity of generalized Daubechies wavelets. Next, in Sect. 4, we prove the necessary conditions stated in Theorem 1.

## Background and preliminary definitions

In this section we recall well-known properties of subdivision schemes. We start by defining convergence and Hölder regularity of non-stationary and, thus, also of stationary subdivision schemes. We would like to distinguish between the following two different types of convergence, both being investigated in the literature on stationary and non-stationary subdivision schemes. We denote by $\ell _\infty (\mathbb {Z}^s)$ the space of all scalar sequences ${\varvec{c}}=\{c(\alpha ), \ \alpha \in \mathbb {Z}^s\}$ indexed by $\mathbb {Z}^s$ and such that $\Vert {\varvec{c}}\Vert _{\ell _\infty }:=\sup _{\alpha \in \mathbb {Z}^s} |c(\alpha )| < \infty .$

### Definition 6

A subdivision scheme $\{S_{\mathbf {a}^{(k)}}, \ k\ge 1\}$ *converges to* $C^\ell$ *limit functions*, if for any initial sequence ${\varvec{c}}\in \ell _\infty (\mathbb {Z}^s)$, there exists the *limit function* $g_{{\varvec{c}}} \in C^\ell (\mathbb {R}^s)$ (which is nonzero for at least one nonzero sequence ${\varvec{c}}$) such that

8 $$\begin{aligned} \lim _{k \rightarrow \infty } \left\| g_{{\varvec{c}}}(M^{-k}\alpha )- S_{\mathbf {a}^{(k)}} S_{\mathbf {a}^{(k-1)}} \ldots S_{\mathbf {a}^{(1)}} {\varvec{c}}(\alpha ) \right\| _{\ell _\infty }= 0. \end{aligned}$$

In the next Definition 9 we consider a stronger type of convergence, the so-called $C^\ell$-convergence of subdivision. Note that both types of convergence coincide in the case $\ell =0$. In Definition 9 we make use of the concept of a test function (see, for example [24]). To define this concept we need to recall the following properties of the test functions.

### Definition 7

Let $\ell \ge 0$. A compactly supported summable function *f* satisfies Strang-Fix conditions of order $\ell +1$, if its Fourier transform $\hat{f}$ satisfies

$$\begin{aligned} \hat{f}(0)=1, \quad D^\mu \hat{f}(\alpha )=0, \quad \alpha \in \mathbb {Z}^s {\setminus } \{0\}, \quad \mu \in \mathbb {N}_0^s, \quad |\mu |< \ell +1. \end{aligned}$$

### Definition 8

A compactly supported $f \in L_\infty (\mathbb {R}^s)$ is stable, if there exists $0<C_1 \le C_2 <\infty$ such that for all ${\varvec{c}}\in \ell _\infty (\mathbb {Z}^s)$,

$$\begin{aligned} C_1 \Vert {\varvec{c}}\Vert _{\ell _\infty } \le \left\| \sum _{\alpha \in \mathbb {Z}^s} c(\alpha )f(\cdot -\alpha ) \right\| _\infty \le C_2\Vert {\varvec{c}}\Vert _{\ell _\infty }. \end{aligned}$$

By [5, p. 24], this type of stability is equivalent to $\ell _\infty$ linear independence of integer shifts of *f*. The function *f* is called *a test function*, if it is sufficiently smooth, compactly supported, stable and satisfies Strang-Fix conditions of order $\ell +1$. Possible examples of the test functions *f* are tensor-product box splines.

### Definition 9

A subdivision scheme $\{S_{\mathbf {a}^{(k)}}, \ k\ge 1\}$ is $C^\ell$ *-convergent*, if for any initial sequence ${\varvec{c}}\in \ell _\infty (\mathbb {Z}^s)$ there exists the limit function $g_{\varvec{c}}\in C^\ell (\mathbb {R}^s)$ such that for any test function $f \in C^\ell (\mathbb {R}^s)$

9 $$\begin{aligned} \lim _{k \rightarrow \infty }\left\| g_{{\varvec{c}}}(\cdot ) - \sum _{\alpha \in \mathbb {Z}^s} S_{\mathbf {a}^{(k)}} S_{\mathbf {a}^{(k-1)}} \ldots S_{\mathbf {a}^{(1)}}\mathbf {c}(\alpha ) f(M^k\cdot - \alpha ) \right\| _{C^\ell }=0. \end{aligned}$$

Note that, for $C^\ell$-convergence, it suffices to check (9) just for one test function *f*. In this paper, we also investigate the Hölder regularity of subdivision limits.

### Definition 10

The *Hölder regularity* of the $C^0$-convergent scheme $\{S_{\mathbf {a}^{(k)}}, \ k\ge 1\}$ is $\alpha =\ell +\zeta$, if $\ell$ is the largest integer such that $g_{{\varvec{c}}} \in C^\ell (\mathbb {R}^s)$ and $\zeta$ is the supremum of $\nu \in [0,1]$ such that

$$\begin{aligned} \max _{\mu \in \mathbb {N}_0^s, |\mu |=\ell } |D^\mu g_{{\varvec{c}}}(x)-D^\mu g_{{\varvec{c}}}(y)| \le |x-y|^\nu , \quad x,y \in \mathbb {R}^s. \end{aligned}$$

We call $\alpha$ the *Hölder exponent* of the limit functions of $\{S_{\mathbf {a}^{(k)}}, \ k\ge 1\}$.

Instead of studying the regularity of all limit functions of a $C^0$-convergent subdivision scheme, one usually restricts the analysis to the so-called *basic limit functions*, which are defined as follows. Let ${\varvec{\delta }}:=\{\delta (\alpha )=\delta _{0,\alpha },\ \alpha \in \mathbb {Z}^s\}$, where $\delta _{0,\alpha }$, $\alpha \in \mathbb {Z}^s$, is the Kronecker delta symbol, i.e., $\delta _{0,0}=1$ and zero otherwise. The compactly supported *basic limit functions* $\phi _k$ generated from the initial sequence ${\varvec{\delta }}$ are given by

$$\begin{aligned} \phi _{k}:=\lim _{\ell \rightarrow \infty } S_{\mathbf {a}^{(k+\ell )}} \ldots S_{\mathbf {a}^{(k+1)}} S_{\mathbf {a}^{(k)}} {\varvec{\delta }}, \quad k \ge 1. \end{aligned}$$

An interesting fact about convergent non-stationary schemes is that the compactly supported *basic limit functions* $\phi _k$ are mutually refinable, i.e., they satisfy the functional equations

10 $$\begin{aligned} \phi _{k}=\sum _{\alpha \in \mathbb {Z}^s} \mathrm{a}^{(k)}(\alpha ) \phi _{k+1}(M\cdot -\alpha ),\quad k \ge 1, \end{aligned}$$

where $\{\mathrm{a}^{(k)}(\alpha ),\ \alpha \in \mathbb {Z}^s\}$ is the *k*-level subdivision mask. We remark that, without loss of generality, to study convergence and regularity of a non-stationary subdivision scheme it suffices to study the continuity and the Hölder regularity of the function $\phi _1$. This fact is shown in the next lemma (see also [58]).

### Lemma 1

Let $\alpha _{\phi _k}$ be the Hölder exponent of $\phi _k$, $k \ge 1$. If $\mathrm{a}^{(k)}(0) \not =0$, then $\alpha _{\phi _k}=\alpha _{\phi _1}$ for all $k \ge 1$.

### Proof

Let $k \ge 1$. Due to (10) and the compact support of the mask $\mathbf {a}^{(k)}$, we have $\alpha _{\phi _{k}} \ge \alpha _{\phi _{k+1}}$ and it suffices to show that $\alpha _{\phi _{k+1}} \ge \alpha _{\phi _{k}}$. To do that we show that, for any compactly supported function *h*, the operator $g=\Phi h=\sum _{\alpha \in \mathbb {Z}^s} a^{(k)}(\alpha )h(\cdot -\alpha )$ preserves the regularity of *h*. Note that, due to $\mathrm{a}^{(k)}(0) \not =0$, its symbol satisfies $a^{(k)}_*(z) \not =0$ in the neighborhood of zero. Thus, the meromorphic function $b^{(k)}_*(z) = 1/a^{(k)}_*(z)$, $z \in \mathbb {C}^s {\setminus } \{0\}$, has the Taylor expansion $b^{(k)}_*(z) = \sum _{\beta \in \mathbb {N}_0^s} b^{(k)}(\beta ) z^\beta$ in the neighborhood of zero. Then, due to $a^{(k)}_*(z)b^{(k)}_*(z)=1$ and by the Cauchy product formula, we get

$$\begin{aligned} \sum _{\beta \in \mathbb {N}_0^s} b^{(k)}(\beta ) g(\cdot -\beta )= & {} \sum _{\beta \in \mathbb {N}_0^s} \sum _{\alpha \in \{0,\ldots ,N\}^s} b^{(k)}(\beta -\alpha ) \mathrm{a}^{(k)}(\alpha ) h(\cdot -\beta )\\= & {} b^{(k)}(0) \mathrm{a}^{(k)}(0) h=h. \end{aligned}$$

Therefore, for the Hölder exponents of *g* and *h* we get $\alpha _h \ge \alpha _g$ and, thus, also $\alpha _{\phi _{k+1}} \ge \alpha _{\phi _{k}}$.$\square$

For our analysis, for the sequence of masks $\{\mathbf {a}^{(k)},\ k\ge 1\}$ supported on $\{0,\ldots ,N\}^s$, we define the so-called transition matrices $T^{(k)}_{\varepsilon }$, $k \ge 1$, $\varepsilon \in E \simeq \{0, \ldots ,m-1\}^s$, as follows. Accordingly to [12], we set

11 $$\begin{aligned} K:=\sum _{r=1}^\infty M^{-r}G,\quad \hbox {where}\quad G:=\{-m,\ldots ,N+1\}^s, \end{aligned}$$

and define the $|K|\times |K|$ matrices

12 $$\begin{aligned} T^{(k)}_{\varepsilon ,\mathbf {a}^{(k)}}:=\left[ \mathrm{a}^{(k)} (\varepsilon +M\alpha -\beta ) \right] _{\alpha , \beta \in K}, \quad \varepsilon \in E. \end{aligned}$$

For simplicity we write $T^{(k)}_{\varepsilon }$ instead of $T^{(k)}_{\varepsilon ,\mathbf {a}^{(k)}}$. If the symbols $\{a^{(k)}_*(z), \ k \ge 1\}$ satisfy sum rules of order $\ell +1$, then the linear operators $T^{(k)}_{\varepsilon }$ have common invariant difference subspaces $V_j \subset \mathbb {R}^{|K|}$, each of which is orthogonal to $\{ [p(\alpha )]_{\alpha \in K} \in \mathbb {R}^{|K|}\ : \ p \in \Pi _n\}$, $n=0, \ldots , j$. The spaces $\Pi _j$ are the spaces of polynomials of total degree less than or equal to $j=0, \ldots ,\ell$. The existence of $V_j$, $j=0,\ldots , \ell$, is guaranteed for $C^\ell$-convergent stationary subdivision schemes and is indeed used for analysis of convergence and regularity in the stationary setting. We refer the reader, for example, to the papers [4, 5, 7, 12, 43, 47] for more details on the structure of $V_j$ and for characterizations of regularity of stationary subdivision schemes in terms of spectral properties of the matrices $T_{\varepsilon , {\mathbf a}}|_{V_j}$, $\varepsilon \in E$. Similarly to (12), these matrices are derived from the stationary mask $\mathbf a$ as follows: define

13 $$\begin{aligned} T_{\varepsilon , {\mathbf a}}:=\left[ \mathrm{a}(\varepsilon +M\alpha -\beta ) \right] _{\alpha , \beta \in K}, \quad \varepsilon \in E, \end{aligned}$$

and determine their restrictions $T_{\varepsilon , {\mathbf a}}|_{V_j}$ to $V_j$. Since, in general, in the non-stationary setting, the existence of such invariant subspaces is not guaranteed by the regularity of the limit functions, in this paper we study non-stationary schemes $\{S_{\mathbf {a}^{(k)}}, \ k\ge 1\}$ whose sequences of masks possess sets ${\mathcal A}$ of limit points, see Definition 4. This allows us, similarly to the stationary setting, to establish a link between the regularity of non-stationary schemes and the spectral properties of the collection of square $|K| \times |K|$ matrices $T_{\varepsilon , \mathbf {a}}$ restricted to $V_j$, $j=0,\ldots ,\ell$. This collection we denote by ${\mathcal T}_{{\mathcal A}}|_{V_j}:=\{T_{\varepsilon ,\mathbf {a}}|_{V_j},\ \varepsilon \in E,\ \mathbf {a}\in {\mathcal A}\}.$

We conclude this section by recalling the notion of the joint spectral radius of a set of square matrices, see [64].

### Definition 11

The joint spectral radius (JSR) of a compact collection of square matrices ${{\mathcal M}}$ is defined by ${ \rho ({{\mathcal M}}):=\lim _{n \rightarrow \infty } \max _{M_{1}, \ldots , M_n \in {\mathcal M}} \Vert \prod _{j=1}^n M_{j} \Vert ^{1/n}.}$

Note that $\rho ({{\mathcal M}})$ is independent of the choice of the matrix norm $\Vert \cdot \Vert$.

## Convergence and Hölder regularity of non-stationary schemes

In this section we derive sufficient conditions for convergence and Hölder regularity of a wide class of non-stationary subdivision schemes. In our proofs we make use of the special structure of the matrices $T_{\varepsilon , \mathbf {a}}$, $\mathbf {a} \in \mathcal A$ in (13), and the matrices $T_{\varepsilon }^{(k)}$ in (12) associated with a sequence of masks $\{\mathbf {a}^{(k)},\ \ k \ge 1\}$. This structure is ensured after a suitable change of basis, which we discuss in Sect. 3.1. In the rest of the paper, we call such a basis *a transformation basis*. In Sect. 3.2, we illustrate two important differences between sum rules and approximate sum rules. In Sect. 3.3, we show that a non-stationary subdivision scheme $\{S_\mathbf {a}^{(k)},\ \ k \ge 1\}$ is convergent if its symbols satisfy approximate sum rules of order 1 and, in addition, its sequence of masks $\{\mathbf {a}^{(k)},\ \ k \ge 1\}$ possesses the set of limit points ${\mathcal A}$ such that $\rho \left( {\mathcal T}_{{\mathcal A}}|_{V_0}\right) <1$. Note that, after an appropriate adaptation of the notation in Sects. 1 and 2, the proof of this convergence result is also valid in the case of a general integer dilation matrix *M*, the spectral radius of whose inverse satisfies $\rho (M^{-1})<1$. In Sect. 3.4, we analyze the Hölder regularity of the basic limit function $\phi _1$ under the assumptions of approximate sum rules of order $\ell +1$, $\ell \ge 0$, and $\rho \left( {\mathcal T}_{{\mathcal A}}|_{V_\ell }\right) <m^{-\ell }$. In Sect. 3.5, we prove Theorem 3 and show that under a certain stability assumption the quantity $\rho \left( {\mathcal T}_{{\mathcal A}}|_{V_\ell }\right)$ determines the exact Hölder exponent of the subdivision limits. This result allows us to prove in Sect. 3.6 a recent conjecture on regularity of Daubechies wavelets stated in [32]. In Sect. 3.6, we also illustrate our results with several examples.

We start by stating important properties of the set ${\mathcal A}$.

### Proposition 1

Let $\ell \ge 0.$ Let ${\mathcal A}$ be the set of limit points of $\{\mathbf {a}^{(k)},\ \ k \ge 1\}$. Assume that $\{a_*^{(k)}(z),\ \ k \ge 1\}$ satisfy approximate sum rules of order $\ell +1$. Then, the symbols associated with the masks in ${\mathcal A}$ satisfy sum rules of order $\ell +1$.

### Proof

The proof follows from Definition 4 and the fact that approximate sum rules in Definition 3 imply that $\lim _{k\rightarrow \infty }\delta _k=\lim _{k\rightarrow \infty }\mu _k=0$.$\square$

Next, we would like to remark that the class of the non-stationary schemes we analyze is not empty.

### Remark 1

In general, for an arbitrary compact set ${\mathcal A}$ of masks, there exists a non-stationary subdivision scheme $\{S_{\mathbf {a}^{(k)}}, \ k \ge 1\}$ with the set of limit points $\mathcal A$. One possible way of constructing $\{S_{\mathbf {a}^{(k)}}, \ k \ge 1\}$ from a given set ${\mathcal A}$ is presented in Example 2.

### Transformation basis

Let $\ell \ge 0$ and $\Pi _j$ be the spaces of polynomials of total degree less than or equal to $j=0, \ldots ,\ell$. If the symbols of the masks ${\mathbf {a}} \in {\mathcal A}$ satisfy sum rules of order $\ell +1$, then the corresponding stationary subdivision operators $S_{\mathbf {a}}$ posses certain polynomial eigensequences $\{p_{\mathbf a }(\alpha ), \ \alpha \in \mathbb {Z}^s\}$, $p_{\mathbf a} \in \Pi _j$, $j =0, \ldots ,\ell$. These polynomial egensequences are possibly different for different $\mathbf {a}$. For each $j=0, \ldots , \ell$, the number $d_{j+1}$ of such eigensequences is equal to the number of monomials $x^\eta$, $\eta \in \mathbb {N}_0^s$, of total degree $|\eta |=j$, see [42, 45]. These eigensequences, written in a vector form with ordering of the entries as in (12), become common left-eigenvectors of the corresponding matrices $T_{\varepsilon , \mathbf {a}}$. There are at least two different ways of constructing the so-called *transformation basis* of $\mathbb {R}^{|K|}$. The approach in [4] makes use of the eigensequences of the stationary subdivision operator. We cannot do that as the eigensequences of $S_{\mathbf a}$, $\mathbf {a} \in {\mathcal A}$, possibly differ for different $\mathbf {a}$. For that reason, we follow the approach in [26, 58, 61], which makes use of the elements in the common invariant subspaces $V_j$ of $T_{\varepsilon , \mathbf {a}}$, $\mathbf {a} \in {\mathcal A}$. The *transformation basis* can be constructed as follows: Take the first unit vector of $\mathbb {R}^{|K|}$ and extend it to a basis of $\mathbb {R}^{|K|}$ by choosing appropriate $d_{j+2}$ vectors from $V_j$, $j=0, \ldots , \ell -1$, and a complete basis of $V_\ell$. Note that, any vector from $V_j$ is constructed to be orthogonal to the polynomial vectors $[p_{\mathbf a}(\alpha )]_{\alpha \in K}$, $p_{\mathbf a} \in \Pi _i$, $i =0, \ldots , j$. We choose $d_{j+2}$ vectors, say $v_{j,\eta }$, from $V_j$ in such a way that they are orthogonal to all but one vector $[\alpha ^\eta ]_{\alpha \in K}$ for the corresponding $\eta \in \mathbb {N}_0^s$ with $|\eta |=j+1$.

This choice of the transformation basis guarantees that the transformed matrices $T_{\varepsilon , \mathbf {a}} \in \mathbb {R}^{|K| \times |K|}$, $\varepsilon \in E$, are block-lower triangular and of the form

14 $$\begin{aligned} \left( \begin{array}{ccccc} 1&{} &{} &{} &{} 0\\ &{} B_2 &{} &{} &{} \\ b_{1,\varepsilon ,\mathbf {a}} &{} &{}\ddots &{} &{}\\ &{} b_{2,\varepsilon ,\mathbf {a}} &{} &{}B_{\ell +1} &{} \\ &{}&{}&{} b_{\ell +1,\varepsilon ,\mathbf {a}}&{} T_{\varepsilon , \mathbf {a}}|_{V_\ell } \end{array} \right) , \end{aligned}$$

where the $d_j \times d_j$ matrices $B_{j}$ are diagonal with diagonal entries equal to $m^{-j+1}$; the matrices $b_{j,\varepsilon ,\mathbf {a}}$ are of size $\left( |K|- \sum _{i=1}^j d_i\right) \times d_j$. Moreover, if $\{a_*^{(k)}(z),\ k\ge 1\}$ satisfy approximate sum rules of order $\ell +1$, then, after the same change of basis, the matrices $T_\varepsilon ^{(k)} \in \mathbb {R}^{|K| \times |K|}$, $\varepsilon \in E$, $k \ge 1$, are sums of a block-lower and a block-upper triangular matrices

15 $$\begin{aligned} \widetilde{T}_\varepsilon ^{(k)}+\Delta _\varepsilon ^{(k)}:=\left( \begin{array}{ccccc} 1&{} &{} &{} &{} 0\\ &{} B_2 &{} &{} &{} \\ b_{1,\varepsilon }^{(k)} &{} &{}\ddots &{} &{}\\ &{} b_{2,\varepsilon }^{(k)} &{} &{}B_{\ell +1} &{} \\ &{}&{}&{} b_{\ell +1,\varepsilon }^{(k)}&{}Q_\varepsilon ^{(k)} \end{array} \right) + \left( \begin{array}{ccccc} c_{1,\varepsilon }^{(k)}&{} \ldots &{} &{} &{}\\ &{} c_{2,\varepsilon }^{(k)}&{} \ldots &{} &{} \\ &{} &{} \ddots &{} &{}\\ &{} &{} &{} c_{\ell +1,\varepsilon }^{(k)} &{} \ldots \\ 0&{}&{}&{} &{} \mathrm{O} \end{array} \right) , \end{aligned}$$

where $b_{j,\varepsilon }^{(k)}$ are of size $\left( |K|- \sum _{i=1}^j d_i\right) \times d_j$; the matrices $c_{j,\varepsilon }^{(k)}$ of size $d_j \times \left( |K|-\sum _{i=1}^{j-1} d_i\right)$; $\mathrm{O}$ is the zero matrix of the same size as $Q^{(k)}_\varepsilon$.

### Sum rules versus approximate sum rules

The following example illustrates two important differences between sum rules and approximate sum rules stated in Definitions 3 and 4, respectively. Firstly, the re-scaling of all symbols of a non-stationary subdivision masks to ensure that $\mu _k=0$, $k \ge 1$, can change the properties of the non-stationary scheme if the sequence $\{\mu _k,\ k\ge 1\}$ is not summable. In other words, in contrast to the stationary case, the properties of $a^{(k)}_*(1)$, $k \ge 1$, are crucial for convergence and regularity analysis of non-stationary schemes. Secondly, even in the univariate case, the existence of the factor $(1+z)$ for all non-stationary symbols $a^{(k)}_*(z)$ and the contractivity of the corresponding difference schemes do not guarantee the convergence of the associated non-stationary scheme, if $\{\mu _k, \ k \ge 1\}$ is not summable.

#### Example 1

Let $s=1,\ M=2$. It is well-known that the convergence of $S_\mathbf {a}$ in the stationary case is equivalent to the fact that the difference (or derived) scheme $S_{\mathbf {b}}$ with the symbol $b_*(z)$ such that

$$\begin{aligned} {a_*}(z)= \left( 1+z \right) {b_*}(z), \quad z \in \mathbb {C}{\setminus } \{0\}, \end{aligned}$$

is zero convergent, i.e, for every $v \in \mathbb {R}^{|K|}$ orthogonal to a constant vector and $\varepsilon _1, \ldots , \varepsilon _k \in \{0,1\}$, the norm $\Vert T_{\varepsilon _1,\mathbf {a}} \ldots T_{\varepsilon _k, \mathbf {a}} v\Vert$ goes to zero as *k* goes to $\infty$. In the non-stationary case, this characterization is no longer valid. Consider the non-stationary scheme with the masks

16 $$\begin{aligned} \mathbf {a}^{(k)}:=\left( 1+\frac{1}{k} \right) \mathbf {a}, \quad k \ge 1. \end{aligned}$$

Note that $\mu _k=\frac{2}{k}$, $\delta _k=0$ and, thus, we can conclude that the non-stationary scheme $\{S_{\mathbf {a}^{(k)}}, \ k \ge 1\}$ does not satisfy approximate sum rules. However, it is asymptotically similar to $S_\mathbf {a}$ and the associated symbols satisfy

$$\begin{aligned} {a_*}^{(k)}(z):= \left( 1+z \right) \left( 1+\frac{1}{k} \right) {b_*}(z),\quad k \ge 1, \quad z \in \mathbb {C}{\setminus } \{0\}. \end{aligned}$$

We show next that the zero convergence of the associated difference schemes with symbols $\left( 1+\frac{1}{k} \right) b_*(z)$ does not imply the convergence of the corresponding non-stationary scheme. Indeed, for $\varepsilon _j \in \{0,1\}$, we get

$$\begin{aligned} \Vert T_{\varepsilon _1}^{(1)} \ldots T_{\varepsilon _k}^{(k)} v \Vert =\prod _{j=1}^k \left( 1+\frac{1}{j}\right) \Vert T_{\varepsilon _1,\mathbf {a}} \ldots T_{\varepsilon _k,\mathbf {a}} v \Vert = (k+1)\Vert T_{\varepsilon _1,\mathbf {a}} \ldots T_{\varepsilon _k,\mathbf {a}} v \Vert . \end{aligned}$$

The convergence of $S_\mathbf {a}$ implies the existence of an operator norm such that

$$\begin{aligned} \Vert T_{\varepsilon _1,\mathbf {a}} \ldots T_{\varepsilon _k,\mathbf {a}} v\Vert \le C \gamma ^k, \quad C>0, \quad \gamma <1. \end{aligned}$$

Therefore, the norm $\Vert T_{\varepsilon _1}^{(1)} \ldots T_{\varepsilon _k}^{(k)} v \Vert$ goes to zero as *k* goes to $\infty$, but the corresponding non-stationary scheme is not convergent. Otherwise, the Fourier-transform of its basic limit function $\phi _1$ would satisfy ${ \hat{\phi }_1(\omega )=\prod _{j=1}^\infty {a}_*^{(j)}(e^{-i2 \pi 2^{-j}\omega }), \ \omega \in \mathbb {R},}$ but

$$\begin{aligned} \hat{\phi }_1(0)= \lim _{k \rightarrow \infty }2 \prod _{j=1}^k \left( 1+\frac{1}{j} \right) {b}_*(1)=\lim _{k \rightarrow \infty }2(k+1){b}_*(1)=\infty . \end{aligned}$$

Note that, if we rescale the masks so that all $\mu _k=0$, $k \ge 1$, we get back the convergent stationary scheme $S_\mathbf {a}$.

### Convergence

We start by recalling that, in the stationary case, for convergence analysis via the joint spectral radius approach one uses the subspace

17 $$\begin{aligned} V_0:=\left\{ v \in \mathbb {R}^{|K|}: \ \sum _{j=1}^{|K|} v_j=0\right\} , \end{aligned}$$

where *K* is defined in (11). This subspace also plays an important role in the proof of the following Theorem 4 that provides sufficient conditions for convergence of a certain big class of non-stationary schemes. In the case $M=mI$, $m \ge 2$, Theorem 4 is an instance of Theorem 2 with $\ell =0$. Note though that in the proof of Theorem 4 we do not assume that $M=mI$, $m \ge 2$, and, thus, we need a more general definition of approximate sum rules of order 1.

#### Definition 12

Let $\Xi =\{ e^{2\pi i M^{-T} \xi } \ : \ \xi \ \hbox {is a coset representative of} \ \mathbb {Z}^s / M^T \mathbb {Z}^s\}\,$. The symbols $\{a^{(k)}_*(z),\ \ k \ge 1\}$ satisfy approximate sum rules of order 1, if the sequences $\left\{ \mu _k,\ k \ge 1 \right\}$ and $\left\{ \delta _k, \ k\ge 1 \right\}$ with

18 $$\begin{aligned} \mu _k:=\left| {a}_*^{(k)}(1)-|\det (M)|\right| \quad \hbox {and}\quad \delta _k:=\max _{\epsilon \in \Xi {\setminus } \{1\}} {| {a}_*^{(k)}(\epsilon )|} \end{aligned}$$

are summable.

In the case of a general dilation matrix, the set *E* is the set of coset representatives $E\simeq \mathbb {Z}^s / M \mathbb {Z}^s$.

#### Theorem 4

Assume that the sequence of symbols $\{ a_*^{(k)}(z), \ k \ge 1\}$ satisfies approximate sum rules of order 1 and $\rho \left( {\mathcal T}_{{\mathcal A}}|_{V_0}\right) <1$, where ${\mathcal A}$ is the set of limit points of $\{ \mathbf {a}^{(k)}, \ k \ge 1\}$. Then the non-stationary scheme $\{ S_{{\mathbf a}^{(k)}}, \ k \ge 1\}$ is $C^0$-convergent.

#### Proof

By [37], the convergence of a non-stationary scheme is equivalent to the convergence of the associated cascade algorithm. Thus, to prove the convergence of the non-stationary scheme $\{ S_{{\mathbf a}^{(k)}}, \ k \ge 1\}$, we show, for $v \in \mathbb {R}^{|K|}$, that the vector-sequence with the elements $T^{(1)}_{\varepsilon _1} \ldots T^{(k)}_{\varepsilon _k} v, \quad k \ge 1$, converges as *k* goes to infinity for every choice of $\varepsilon _1, \ldots \varepsilon _k, \in E$.

Due to Proposition 1, each $\mathbf {a}\in {\mathcal A}$ satisfies sum rules of order 1. Therefore, by Sect. 3.1, the vector $\left( 1 \ 0 \ldots 0 \right)$ is a common left eigenvector of all matrices

$$\begin{aligned} T_{\varepsilon , \mathbf {a}}= \left( \begin{array}{lcr} 1 &{} 0\cdots 0 &{} \\ &{} &{} \\ b_{\varepsilon ,\mathbf {a}} &{} \quad T_{\varepsilon ,\mathbf {a}}|_{V_0} \quad &{} \\ &{} &{} \\ \end{array} \right) , \quad \varepsilon \in E, \quad \mathbf {a} \in {\mathcal A}. \end{aligned}$$

Due to the assumption of approximate sum rules of order 1, by Sect. 3.1, we have

19 $$\begin{aligned} T^{(k)}_\varepsilon = \widetilde{T}^{(k)}_\varepsilon + \Delta ^{(k)}_\varepsilon , \quad \varepsilon \in E, \quad k \ge 1, \end{aligned}$$

with

$$\begin{aligned} \widetilde{T}^{(k)}_\varepsilon = \left( \begin{array}{lcr} 1 &{} 0\cdots 0 &{} \\ &{} &{} \\ b^{(k)}_{\varepsilon } &{} \quad Q^{(k)}_{\varepsilon } \quad &{} \\ &{} &{} \\ \end{array} \right) , \qquad \Delta ^{(k)}_\varepsilon = \left( \begin{array}{ccc} &{} c^{(k)}_\varepsilon &{} \\ &{} O &{} \\ \end{array} \right) . \end{aligned}$$

Thus, the canonical row unit vector $\left( 1 \ 0 \ldots 0 \right)$ is a *quasi*-common left-eigenvector of the operators $T^{(k)}_{\varepsilon }$, $\varepsilon \in E$, i.e. $\left( 1 \ 0 \ldots 0 \right) T^{(k)}_{\varepsilon }=\left( 1 \ 0 \ldots 0 \right) + c^{(k)}_\varepsilon$, where the row vector $c^{(k)}_\varepsilon$ vanishes as *k* tends to infinity and the corresponding sequence of norms $\{\Vert \Delta ^{(k)}_\varepsilon \Vert , \ k \ge 1\}$ is summable. Moreover, $b^{(k)}_\varepsilon$ and $Q^{(k)}_\varepsilon$ converge by subsequences as *k* goes to infinity to $b_{\varepsilon , \mathbf {a}}$ and $T_{\varepsilon ,\mathbf {a}}|_{V_0}$ for some $\mathbf {a} \in {\mathcal A}$, respectively.

By assumption $\rho \left( \{ T_{\varepsilon , \mathbf {a}}|_{V_0}, \ \varepsilon \in E, \mathbf {a} \in {\mathcal A}\} \right) < 1$. Thus, the existence of the operator norm of $\{ T_{\varepsilon , \mathbf {a}}|_{V_0}, \ \varepsilon \in E, \mathbf {a} \in {\mathcal A}\}$ and the continuity of the joint spectral radius imply that there exists $\bar{k}$ such that $\rho \left( \{ Q^{(k)}_\varepsilon , \varepsilon \in E, k \ge \bar{k}\} \right) < 1$. This implies that for all vectors $v \in \mathbb {R}^{|K|}$, the product $\widetilde{T}^{(1)}_{\varepsilon _1} \ldots \widetilde{T}^{(k)}_{\varepsilon _k} v$ converges as *k* goes to infinity for every choice of $\varepsilon _1,\ldots ,\varepsilon _k \in E$.

By well-known results on the joint spectral radius of block triangular families of matrices (see e.g. [1]), we obtain that $\rho \left( \{ \widetilde{T}^{(k)}_\varepsilon , \ \varepsilon \in E, k \ge \bar{k}\} \right) = 1.$ Moreover, the family of matrices $\{ \widetilde{T}^{(k)}_\varepsilon , \ \varepsilon \in E, k \ge \bar{k}\}$ is non-defective (see e.g. [40]), thus by [1, 64], there exists an operator norm $\Vert \cdot \Vert$ such that

20 $$\begin{aligned} \Vert \widetilde{T}^{(k)}_\varepsilon \Vert \le 1 \quad \text{ for } \text{ all } \quad \varepsilon \in E, \quad k \ge \bar{k}. \end{aligned}$$

Due to our assumption that the approximate sum rules of order 1 are satisfied, we also have

21 $$\begin{aligned} \Vert \Delta ^{(k)}_\varepsilon \Vert \le C \delta _{k} \quad \text{ where } \quad \sum \limits _{k=1}^{\infty } \delta _{k}< \infty , \end{aligned}$$

and *C* is a constant which does not depend on *k*.

Next, for $n,\ell \in \mathbb {N}$, we observe that

$$\begin{aligned} T^{(n)}_{\varepsilon _n} \ldots T^{(n+\ell )}_{\varepsilon _{n+\ell }} = \left( \widetilde{T}^{(n)}_{\varepsilon _n} + \Delta ^{(n)}_{\varepsilon _n} \right) \ldots \left( \widetilde{T}^{(n+\ell )}_{\varepsilon _{n+\ell }} + \Delta ^{(n+\ell )}_{\varepsilon _{n+\ell }} \right) = \widetilde{T}^{(n)}_{\varepsilon _n} \ldots \widetilde{T}^{(n+\ell )}_{\varepsilon _{n+\ell }} + R_{n,\ell }, \end{aligned}$$

where $R_{n,\ell }$ is obtained by expanding all the products. From (20), (21) we get $\lim \nolimits _{n \rightarrow \infty } R_{n,\infty } = \mathrm{O}$ implying convergence of $\prod \nolimits _{j=1}^{k} T^{(j)}_{\varepsilon _j} v$ as $k \rightarrow \infty$. The reasoning for $\lim \nolimits _{n \rightarrow \infty } R_{n,\infty } = \mathrm{O}$ is as follows

$$\begin{aligned} \Vert R_{n,\infty }\Vert \le \sum _{j=1}^\infty \left( \sum _{k=n}^{\infty } \delta _k \right) ^j= \sum _{j=0}^\infty \left( \sum _{k=n}^{\infty } \delta _k \right) ^j -1 = \displaystyle \sum _{k=n}^{\infty } \delta _k \left( 1-\displaystyle \sum _{k=n}^{\infty } \delta _k \right) ^{-1}. \end{aligned}$$

$$\square $$

#### Corollary 2

Let $\{ S_{{\mathbf a}^{(k)}}, \ k \ge 1\}$ be a $C^0$-convergent subdivision scheme with the set of limit points ${\mathcal A}$ such that $\rho ({\mathcal T}_{\mathcal A}|_{V_0})<1$. Then any other asymptotically similar non-stationary scheme $\{ S_{{\mathbf b}^{(k)}}, \ k \ge 1\}$ satisfying approximate sum rules of order 1 is $C^0$-convergent.

We would like to remark that Theorem 4 generalizes [18, Theorem 10] dealing with the binary univariate case under the assumption that the non-stationary scheme reproduces constants. Theorem 4 is also a generalization of the corresponding results in [34, 36] that require that stationary and non-stationary schemes are asymptotically equivalent.

### $C^\ell$-convergence and Hölder regularity

In this section we prove Theorem 2 stated in the Introduction, i.e. we derive sufficient conditions for Hölder regularity of non-stationary multivariate subdivision schemes. Note that Theorem 2 with $\ell =0$ also implies the convergence of the corresponding non-stationary scheme. We, nevertheless, gave the proof of $C^0$-convergence separately in Theorem 4, see Sect. 3.3, to emphasize that it is not affected by the choice of the dilation matrix *M*, whereas our proof of $C^\ell$-convergence in this section does depend on the choice of $M=mI$, $m \ge 2$. The proof of Theorem 2 is long, thus, in Sect. 3.4.1, we present several crucial auxiliary results and then prove this theorem in Sect. 3.4.2.

#### Auxiliary results

In the proof of Theorem 2 we make use of the summable sequence $\{\eta _k, \ k \ge 0\}$ which we define next. Note first that under the assumption $\rho ({\mathcal T}_{\mathcal A}|_{V_\ell })<m^{-\ell }$ of Theorem 2, there exist $\gamma \in (0,m^{-\ell })$ and $\bar{k}$ such that

22 $$\begin{aligned} \Vert Q_\varepsilon ^{(k)}\Vert< \gamma < m^{-\ell }, \quad \varepsilon \in E, \quad k \ge \bar{k}, \end{aligned}$$

where $Q_\varepsilon ^{(k)}$ are sub-matrices of the matrices $\widetilde{T}_\varepsilon ^{(k)}$ in (15). This property of $Q_\varepsilon ^{(k)}$ is guaranteed by [1, 64] and the convergence of $\{Q_\varepsilon ^{(k)}, \ k \ge 1\}$ to $T_{\epsilon ,\mathbf {a}}|_{V_j}$. Furthermore, by approximate sum rules of order $\ell +1$ (Definition 3), the sequence $\{\sigma _0:=1, \ \sigma _k=m^{k\ell }\delta _k, \ k \ge 1\}$ is summable and so is the sequence $\{\eta _k, \ k \ge 0\}$ with

23 $$\begin{aligned} \eta _k:=\sum _{j=0}^k \sigma _j q^{k-j}, \quad q:=m^{\ell } \gamma . \end{aligned}$$

Indeed, since $q < 1$, we have

24 $$\begin{aligned} \sum _{k=0}^{\infty }\eta _k \, = \, \sum _{j=0}^{\infty } \sigma _j \sum _{n=0}^{\infty } q^n \, = \, \frac{1}{1-q}\sum _{j=0}^{\infty } \sigma _j \, < \, \infty . \end{aligned}$$

In the following Lemma 2 we estimate the asymptotic behavior of the matrix products

25 $$\begin{aligned} P_k \, = \ R_1\, \ldots \, R_k, \quad k \ge 1, \end{aligned}$$

where, for some non-negative real number *c*, the $(\ell +2) \times (\ell +2)$ matrices $R_j$ are defined by

26 $$\begin{aligned} R_j:=\ \left( \begin{array}{cccccc} 1 +\sigma _j m^{-\ell j} &{} \sigma _j m^{-\ell j} &{} \sigma _j m^{-\ell j} &{} \ldots &{} \sigma _j m^{-\ell j} &{} \sigma _j m^{-\ell j} \\ c &{} m^{-1} + \sigma _j m^{-(\ell -1)j}&{} \sigma _j m^{-(\ell -1)j} &{} \ldots &{}\sigma _j m^{-(\ell -1)j} &{} \sigma _j m^{-(\ell -1)j} \\ c &{} c &{} {} &{} &{} \vdots &{} \vdots \\ \vdots &{} \vdots &{} \ddots &{} &{} \vdots &{} \vdots \\ c &{} \ldots &{} c &{} &{} \sigma _j m^{-j} &{} \sigma _j m^{-j} \\ c &{} \ldots &{} c &{} \ddots &{} m^{-\ell } + \sigma _j &{} \sigma _j \\ c &{} \ldots &{} c &{} \ldots &{} c &{} \gamma \end{array} \right) .\nonumber \\ \end{aligned}$$

In particular, in Lemma 2, we show that any norm of the *r*th column of the matrix product $P_k$ is bounded uniformly over $k \ge 1$, i.e. that any norm of the column $P_k e_r$, with $e_r$ being the standard *r*th unit vector, is bounded uniformly over $k \ge 1$.

##### Lemma 2

For every $$k \in \mathbb {N}$$

$$\begin{aligned} \Vert P_ke_r\Vert _1=\left\{ \begin{array}{ll} \mathcal{O}(m^{-(r-1)k}), &{}\quad r = 1, \ldots , \ell +1, \\ \mathcal{O}( m^{(\ell +1)}\, m^{-\ell k}), &{}\quad r=\ell +2. \end{array} \right. \end{aligned}$$

##### Proof

For simplicity of presentation we consider the case of $M=2I$, i.e $m=2$. Let $C_1$ be the smallest constant such that for each $r = 1, \ldots , \ell +1$, the *r*th column of $R_1$ does not exceed $C_1 2^{-(r-1)}$, and the $(\ell +2)$nd column does not exceed $C_1 2 \eta _1$. We show by induction on *k* that the sum of the *r*th column entries of $P_k$ does not exceed $C_k\, 2^{-(r-1)k}$, $r = 1, \ldots , \ell +1$, or $C_k\, 2^{(\ell +1)}\, 2^{-\ell k}\, \eta _k$, $r=\ell +2$, where

$$\begin{aligned} C_k \ = \ C_1 \prod _{j=2}^{k} \Bigl (1 + 2^{\, \ell +1} \sigma _j \, + \, c \, 2^{\, \ell } 2^{\,2-j} + c 2^{\, 2\ell +1}\, \, \eta _{j-1}\Bigr ), \quad k \ge 2. \end{aligned}$$

Due to

27 $$\begin{aligned} C_{k} \ = \ C_{k-1}\Bigl (1 + 2^{\, \ell +1} \sigma _k \, + \, c \, 2^{\, \ell } 2^{\,2-k} + c 2^{\, 2\ell +1}\, \, \eta _{k-1}\Bigr ), \quad k \ge 2, \end{aligned}$$

the sequence $\{C_k, \ k \ge 1\}$ increases and converges to

28 $$\begin{aligned} \tilde{C} \ := \ C_1\, \prod _{j=2}^{\infty } \, \Bigl (1 + 2^{\, \ell +1} \sigma _j \, + \, c \, 2^{\, \ell } 2^{\,2-j} + c 2^{\, 2\ell +1}\, \, \eta _{j-1}\Bigr ). \end{aligned}$$

Since the sums $\sum \nolimits _{j=1}^{\infty } \sigma _j \, , \, \sum \nolimits _{j=1}^{\infty } 2^{\, 2 - j}$, and $\sum \nolimits _{j=1}^{\infty } \eta _{j-1}$ are all finite, the infinite product in (28) converges. By induction assumption, we have $\Vert P_{k-1} e_j\bigr \Vert _1 \le C_{k-1}2^{-(j-1)(k-1)}$, for $j=1, \ldots , \ell +1$, and $\Vert P_{k-1} e_{\ell +2}\bigr \Vert _1 \le C_{k-1}2^{\ell +1}\, 2^{-\ell (k-1)}\eta _{k-1}$. Since the *r*th column of $P_k$ is $P_ke_r$, where $e_r$ is the *r*th basis vector of $\mathbb {R}^{\ell +2}$, we have

$$\begin{aligned} \Bigl \Vert P_ke_r \Bigr \Vert _{1} \ = \ \Bigl \Vert P_{k-1} R_k e_r \Bigr \Vert _{1} \ \le \ \sum _{j=1}^{\ell +2} \, \Bigl \Vert P_{k-1} e_j\Bigr \Vert _1 \bigl ( R_k\bigr )_{jr}\, . \end{aligned}$$

Thus,

29 $$\begin{aligned} \bigl \Vert P_{k}e_r \bigr \Vert _1 \ \le \ \sum _{j=1}^{\ell +2} \, \Bigl \Vert P_{k-1} e_j\Bigr \Vert _1 \bigl ( R_k\bigr )_{jr}\, . \end{aligned}$$

Next we consider the following three cases.

*Case 1*: $r=1$. The first column of the matrix $R_k$ is $(1+ \sigma _k 2^{-\ell k}, c, \ldots , c)^T$. By induction assumption and due to $\sum _{j=2}^{\ell +1} 2^{-(j-1)(k-1)}=0$ for $\ell =0$ and (27), the estimate (29) yields

$$\begin{aligned} \bigl \Vert P_{k}e_1 \bigr \Vert _1\le & {} C_{k-1}\, \Bigl ( 1 + \sigma _k 2^{-\ell k} + \, c \sum _{j=2}^{\ell +1} 2^{-(j-1)(k-1)}\, + \, c\, 2^{\ell +1}\, 2^{-\ell (k-1)}\eta _{k-1} \Bigr ) \\\le & {} C_{k-1}\, \Bigl ( 1 + \sigma _k 2^{-\ell k} + \, 2\, c\, 2^{-(k-1)}\, + \, c\, 2^{\ell +1}\, \eta _{k-1} \Bigr ) \ \le \ C_k. \end{aligned}$$

*Case 2*: $2 \le r\le \ell +1$. The *r*th column of the matrix $R_k$ is

$$\begin{aligned} (R_k)_r \ = \ \bigl (\sigma _k2^{-\ell k}, \ldots , \sigma _k 2^{-(\ell -(r-2))\, k}, 2^{-(r-1)}+ \sigma _k 2^{-(\ell -(r-1))\, k}, c, \ldots , c \bigr )^T. \end{aligned}$$

By induction assumption the estimate in (29) becomes

$$\begin{aligned} \bigl \Vert P_{k}e_r \bigr \Vert _1\le & {} C_{k-1}\, \left( \sigma _k \sum _{j=1}^{r-1} 2^{-(\ell +1-j)k}2^{-(j-1)(k-1)} \, + \, \bigl ( 2^{-(r-1)} + \sigma _k 2^{-(\ell +1 - r)k}\bigr ) 2^{-(r-1)(k-1)}\right. \\&\qquad \quad \qquad + \,\left. c \sum _{j=r+1}^{\ell +1} 2^{-(j-1)(k-1)} \, + \, c \, 2^{\, \ell +1} 2^{-\ell (k-1)}\, \, \eta _{k-1}\, \right) \\\le & {} C_{k-1}\, \left( \sigma _k 2^{-\ell k}\sum _{j=1}^{r} 2^{j-1} \, + \, 2^{-(r-1)k} \, + \, 2\, c \, 2^{-r(k-1)}\, + \, c\, 2^{\, \ell +1} 2^{-(r-1)(k-1)} \, \, \eta _{k-1}\, \right) \\\le & {} C_{k-1}\, \Bigl ( \sigma _k 2^{-(r-1)k} 2^{r} \, + \, 2^{-(r-1)k} \, + \, c \, 2^{r+1}\, 2^{-(r-1)k} 2^{-k}\, + \, c\, 2^{-(r-1)k} 2^{\, \ell +r}\, \, \eta _{k-1}\, \Bigr )\\\le & {} C_{k-1}\, 2^{-(r-1)k}\Bigl (1 + 2^{\, \ell +1} \eta _k \, + \, c \, 2^{\, \ell } 2^{\,2-k} + c\, 2^{\, 2\ell +1}\, \, \eta _{k-1}\Bigr ) \le \ C_{k}\, 2^{-(r-1)k}\, . \end{aligned}$$

*Case 3*: $r = \ell +2$. The last column of the matrix $R_k$ is $\bigl (\sigma _k2^{-\ell k}, \ldots , \sigma _k2^{-k}, \sigma _k , \gamma \bigr )^T$. Note that by definition of $\eta _k$ we have $\eta _k - \sigma _k = \eta _{k-1}$, and recall that $\gamma < 2^{-\ell }$. Then, by induction assumption, we get

$$\begin{aligned} \bigl \Vert P_k e_{\ell +2} \bigr \Vert _1 \,\le & {} \, C_{k-1}\, \left( \sigma _k \sum _{j=1}^{\ell +1} 2^{-(\ell +1-j)k}2^{-(j-1)(k-1)} \, + \, 2^{\, \ell +1}2^{-\ell (k-1)}\, \gamma \, \eta _{k-1} \right) \\= & {} C_{k-1}\, \left( \sigma _k 2^{-\ell k}\sum _{j=1}^{\ell +1} 2^{j-1} \, + \, 2^{\, \ell +1} 2^{-\ell k} \, \bigl (\eta _k - \sigma _k \bigr )\, \right) \\\le & {} C_{k-1}\, \left( \sigma _k 2^{-\ell k} 2^{\, \ell +1}\, + \, 2^{\, \ell +1} 2^{-\ell k} \, \eta _k\, - \, 2^{\, \ell +1}\, 2^{-\ell k} \sigma _k \right) \\= & {} \, C_{k-1} 2^{\, \ell +1} 2^{-\ell k} \, \eta _k\, \le \, C_k 2^{\, \ell +1} 2^{-\ell k} \, \eta _k. \end{aligned}$$

$$\square $$

The estimates in Lemma 2 allow us to estimate the norms of the columns of the matrix products $T_{\varepsilon _1}^{(1)} \ldots T_{\varepsilon _k}^{(k)}$, $\varepsilon _1, \ldots , \varepsilon _k \in E$.

##### Lemma 3

Let $\varepsilon _1, \ldots , \varepsilon _k \in E$, $\ell \ge 0$. Assume that the symbols of $\{S_{{\mathbf a}^{(k)}}, \ k \ge 1\}$ satisfy approximate sum rules of order $\ell +1$ and $\rho ({\mathcal T}_{\mathcal A}|_{V_\ell })< m^{-\ell }$. Then the norms of the columns of $T_{\varepsilon _1}^{(1)} \ldots T_{\varepsilon _k}^{(k)}$ with indices $1+\sum _{j=1}^{r-1} d_j, \ldots , \sum _{j=1}^{r} d_j$ are equal to $\mathcal{O}(m^{-(r-1)k})$ for $r=1, \ldots , \ell +1$. The norms of the other columns of this matrix product are equal to $\mathcal{O}(m^{-\ell k} \eta _k)$.

##### Proof

Let $\varepsilon \in E$. Under the assumptions of Theorem 2, the matrices $\widetilde{T}_\varepsilon ^{(k)}$ in (15) have the following properties: the matrix sequences $\{b_{j,\varepsilon }^{(k)}, \ k \ge 1\}$ and $\{Q_{\varepsilon }^{(k)}, \ k \ge 1\}$ converge by subsequences as *k* goes to $\infty$, respectively, to $b_{j,\varepsilon ,\mathbf {a}}$ and $T_{\varepsilon ,\mathbf {a}}|_{V_\ell }$ for some $\mathbf {a} \in \mathcal A$; there exists $c>0$ such that all the norms $\Vert b_{j,\varepsilon ,\mathbf {a}}\Vert _\infty \le c < \infty$; the estimate in (22) holds for $0< \gamma < m^{-\ell }$ and for some matrix norm $\Vert \cdot \Vert _{ext}$. Furthermore, approximate sum rules of order $\ell +1$ and the definition of $\sigma _k$ imply that the entries of the matrices $c_{j,\varepsilon }^{(k)}$, $j=1, \ldots , \ell +1$, are bounded by $\sigma _k m^{-(\ell +1-j)k}$. Next, let $L_0=0$ and $L_i= \sum _{j=1}^{i} d_j$, $i=1, \ldots , \ell +1$, with $d_j$ defined in Sect. 3.1. Set $L=L_{\ell +1}$ and write a vector $v=(v_1, \ldots , v_{|K|})^T \in \mathbb {R}^{|K|}$ as

$$\begin{aligned} v=\left( v^{[1]}, v^{[2]}, \ldots , v^{[\ell +1]}, v^{[\ell +2]} \right) \end{aligned}$$

with

$$\begin{aligned} v^{[i]}:=(v_{L_{i-1}+1}, \ldots , v_{L_{i}})^T,\ i=1,\ldots ,\ell +1,\quad v^{[\ell +2]}:=(v_{L+1},\ldots , v_{|K|})^T. \end{aligned}$$

Consider the vector norm ${ \Vert v\Vert :=\sum _{i=1}^{\ell +1} \Vert v^{[i]}\Vert _\infty + \Vert v^{[\ell +2]}\Vert _{ext}, \quad v \in \mathbb {R}^{|K|}.}$ Then

$$\begin{aligned} \Vert T_\varepsilon ^{(k)} v\Vert \le \Vert R_k \tilde{v}\Vert , \quad \tilde{v}=\left( \Vert v^{[1]}\Vert _\infty , \ldots , \Vert v^{[\ell +1]}\Vert _\infty , \Vert v^{[\ell +2]}\Vert _{ext}\right) \in \mathbb {R}^{\ell +2}, \end{aligned}$$

where $R_k$ is given in (26). Analogously, we get

$$\begin{aligned} \Vert T^{(1)}_{\varepsilon _1} \ldots T^{(k)}_{\varepsilon _k} v\Vert \le \Vert R_1 \ldots R_k \tilde{v}\Vert . \end{aligned}$$

The claim follows by Lemma 2.$\square$

#### Proof of Theorem 2

The proof of Theorem 2 is long, so we split it into two parts: Propositions 2 and 3. In the first part of the proof, given in Proposition 2, we show that the assumptions of Theorem 2 are indeed sufficient for the $C^\ell$-convergence of non-stationary schemes. In particular, we let $f \in C^\ell (\mathbb {R}^s)$ be compactly supported, stable and refinable with respect to the dilation matrix $M=mI$ and the mask $\mathbf {e} \in \ell _0(\mathbb {Z}^s)$. Then, for every $j=0, \ldots ,\ell$ and for every $\nu \in \mathbb {N}_0^s$, $|\nu |=j$, we consider the sequence $\{D^\nu f_k, \ k \ge 1\}$, where for $f_k:= {\mathcal T}^{(1)} \ldots {\mathcal T}^{(k)}f$

30 $$\begin{aligned} D^\nu f_k=m^{jk} {\mathcal T}^{(1)} \ldots {\mathcal T}^{(k)} D^\nu f, \quad {\mathcal T}^{(k)} f=\displaystyle \sum _{\alpha \in \mathbb {Z}^s} \mathrm{a}^{(k)}(\alpha ) f(M\cdot -\alpha ), \end{aligned}$$

i.e. ${\mathcal T}^{(k)}$ is the transition operator associated with the mask $\mathbf{a}^{(k)}$, and show that $\{D^\nu f_k, \ k \ge 1\}$ converges uniformly to the $\nu$-th partial derivative of $\phi _1$.

##### Proposition 2

Let $\ell \ge 0$. Assume that the symbols of $\{ S_{{\mathbf a}^{(k)}}, \ k \ge 1\}$ satisfy approximate sum rules of order $\ell +1$ and $\rho ({\mathcal T}_{\mathcal A}|_{V_\ell })<m^{-\ell }$. Then, for every $j=0, \ldots ,\ell$ and for every $\nu \in \mathbb {N}_0^s$, $|\nu |=j$, the sequence $\{D^\nu f_k, \ k \ge 1\}$ in (30) converges uniformly to the $\nu$-th partial derivative of $\phi _1$. Moreover, there exists a constant $C>0$ independent of *k* such that for $\eta _n$ as in (23) we have

31 $$\begin{aligned} \Vert D^\nu f_k-D^\nu \phi _1\Vert _{\infty } \le C \sum _{n=k}^\infty \eta _n, \quad |\nu |=\ell , \quad k \ge 1. \end{aligned}$$

##### Proof

Note that, by [12, p. 137], the function *f* in (30) is an appropriate starting function for the cascade algorithm. Moreover, by [44, Theorem 6.3], the assumptions on *f* imply that *f* satisfies Strang-Fix conditions of order $\ell +1$, i.e. its Fourier transform $\hat{f}$ satisfies

$$\begin{aligned} \hat{f}(0)=1, \quad D^\mu \hat{f}(\alpha )=0, \quad \alpha \in \mathbb {Z}^s {\setminus } \{0\}, \quad \mu \in \mathbb {N}_0^s, \quad |\mu |< \ell +1. \end{aligned}$$

Consequently, its derivatives $D^\nu f$, $\nu \in \mathbb {N}_0^s$, $|\nu |=j$, $j=1, \ldots ,\ell$, satisfy

$$\begin{aligned} D^\mu \widehat{( D^\nu f)}(\alpha )=0, \quad \alpha \in \mathbb {Z}^s, \quad \mu \in \mathbb {N}_0^s, \quad |\mu |< j. \end{aligned}$$

Thus, by the Poisson summation formula, we get

32 $$\begin{aligned} \sum _{\alpha \in \mathbb {Z}^s} p(\alpha ) D^\nu f(x-\alpha )=0, \quad x \in \mathbb {R}^s, \end{aligned}$$

for all polynomial sequences $\{p(\alpha ), \ \alpha \in \mathbb {Z}^s\}$, $p \in \Pi _j$. Note that we can chose *f* such that $\hbox {supp} f \cap \mathbb {Z}^s \subset K$. Then, the properties (32) of $D^\nu f$ imply that, after the transformation discussed in Sect. 3.1, the first $\sum _{i=1}^{j} d_i$ entries of the vectors

$$\begin{aligned} v(x):=\left( D^\nu f(x+\alpha )\right) _{\alpha \in K}, \quad x \in [0,1]^s, \quad |\nu |=j, \quad j=1, \ldots ,\ell , \end{aligned}$$

are equal to zero. Note that the ordering of the entries in *v*(*x*) corresponds to the ordering of the columns of $T_\varepsilon ^{(k)}$ defined in (12). By Theorem 4, the limit functions of the non-stationary scheme are $C^0(\mathbb {R}^s)$, i.e. the sequence $\{f_k, \ k\ge 1\}$ is a uniformly convergent Cauchy sequence. Similarly to the stationary case, to show that the non-stationary scheme is $C^j$-convergent, $j=1,\ldots ,\ell$, we need to study the uniform convergence of the sequences $\{D^\nu f_k, \ k\ge 1\}$ for all $\nu \in \mathbb {N}_0^s$, $|\nu |=j$. Equivalently, for every choice of $\varepsilon _1, \ldots \varepsilon _k \in E$, need to study the convergence of the vector-sequences $\{m^{j k} T^{(1)}_{\varepsilon _1} \ldots T^{(k)}_{\varepsilon _k} w, \ k \ge 1\}$, where $T_\varepsilon ^{(k)}$ are defined from $\{{\mathbf a}^{(k)}, \ k \ge 1\}$ and the vector $w \in \mathbb {R}^{|K|}$ is arbitrary and such that its first $\sum _{i=1}^{j} d_i$ entries are zero. Lemma 3, the structure of *w* and the summability of $\{\eta _k, \ k \ge 1\}$ imply the convergence of the vector-sequences $\{m^{j k} T^{(1)}_{\varepsilon _1} \ldots T^{(k)}_{\varepsilon _k} w, \ k \ge 1\}$ for $j=1, \ldots , \ell$. Thus, the non-stationary scheme is $C^\ell$-convergent.

We prove next the estimate (31). Let $\nu \in \mathbb {N}_0^s$, $|\nu |=\ell$. Due to $\phi _1=\lim _{k \rightarrow \infty } {\mathcal T}^{(1)} \ldots {\mathcal T}^{(k)} f$ and by the assumption of refinability of *f*, i.e. $f={\mathcal T}f=\sum _{\alpha \in \mathbb {Z}^s} \mathrm{e}(\alpha )f(Mx-\alpha )$, we have

$$\begin{aligned} \Vert D^\nu f_k-D^\nu \phi _1\Vert _{\infty }\le & {} \sum _{n=k}^\infty \Vert D^\nu f_{n+1}- D^\nu f_{n} \Vert _{\infty } \\= & {} \sum _{n=k}^\infty m^{\ell (n+1)} \Vert {\mathcal T}^{(1)} \ldots {\mathcal T}^{(n)} \left( {\mathcal T}^{(n+1)}-{\mathcal T}\right) (D^\nu f)(M^{-(n+1)} \cdot )\Vert _{\infty }. \end{aligned}$$

As above, to estimate the norms $\Vert {\mathcal T}^{(1)} \ldots {\mathcal T}^{(n)} \left( {\mathcal T}^{(n+1)}-{\mathcal T}\right) (D^\nu f)(M^{-(n+1)} \cdot )\Vert _{\infty }$, we need to estimate the vector-norms of

$$\begin{aligned} T_{\varepsilon _1}^{(1)} \ldots T^{(n)}_{\varepsilon _{n}} \left( T_{\varepsilon _{n+1}}^{(n+1)}-T_{\varepsilon _{n+1}, \mathbf {e}} \right) w, \end{aligned}$$

where $|K| \times |K|$ matrices $T_{\varepsilon ,\mathbf {e}}$, $\varepsilon \in E$, are derived from the mask $\mathbf {e}$, see (14), and the first $\sum _{j=1}^{\ell +1} d_j$ entries of the vector $w \in \mathbb {R}^{|K|}$ are zero. By assumption, there exists a constant $\beta >0$ such that the entries of all $b_{j,\varepsilon ,\mathbf {e}}$ and $b^{(k)}_{j,\varepsilon }$ are less than $\beta$ in the absolute value. The approximate sum rules of order $\ell +1$ imply that the absolute values of the entries of the vectors $\left( T_{\varepsilon _n}^{(n+1)}-T_{\varepsilon _n,\mathbf {e}} \right) w$ with indices $1+\sum _{j=1}^r d_j, \ldots , \sum _{j=1}^{r+1} d_j$, $r=0, \ldots , \ell$, are bounded respectively by $\sigma _{n+1}m^{-(\ell -r)(n+1)}$. All other entries are bounded by $2\beta$. Thus, by Lemma 3, we get that the entries of the vectors $m^{\ell (n+1)} T_{\varepsilon _1}^{(1)} \ldots T^{(n)}_{\varepsilon _{n}} \left( T_{\varepsilon _{n+1}}^{(n+1)}-T_{\varepsilon _{n+1}, \mathbf {e}} \right) w$ with indices $1+\sum _{j=1}^r d_j, \ldots , \sum _{j=1}^{r+1} d_j$, $r=0, \ldots , \ell$, are equal to $\mathcal{O} (\sigma _{n+1})$, all other entries are equal to $\mathcal{O}(\eta _n)$. Therefore, by definition of $\{\eta _k, \ k \ge 1\}$ in (23), we get

$$\begin{aligned} \Vert D^\nu f_k- D^\nu \phi _1\Vert _\infty \le C \sum _{n=k}^\infty \eta _n, \quad k \ge 1, \end{aligned}$$

for some $C>0$ independent of *k*.$\square$

The second part of the proof of Theorem 2 is given in Proposition 3 which yields the desired estimate for the Hölder regularity $\alpha$ of the scheme $\{ S_{{\mathbf a}^{(k)}}, \ k \ge 1\}$.

##### Proposition 3

Let $k \ge 1$, $h \in \mathbb {R}^s$, $m^{-(k+1)}< \Vert h\Vert _\infty \le m^{-k}$ and $\ell \ge 0$. Assume that the symbols of $\{ S_{{\mathbf a}^{(k)}}, \ k \ge 1\}$ satisfy approximate sum rules of order $\ell +1$ and $\rho ({\mathcal T}_{\mathcal A}|_{V_\ell })<m^{-\ell }$. Then there exists a constant $C>0$ independent of *k* such that, for $\eta _n$ as in (23), we have

33 $$\begin{aligned} \Vert D^\nu \phi _1(\cdot +h)-D^\nu \phi _1(\cdot )\Vert _\infty \le C \sum _{n=k}^\infty \eta _n, \quad \nu \in \mathbb {N}_0^s, \quad |\nu |=\ell . \end{aligned}$$

Moreover, the Hölder exponent $\alpha$ of $\phi _1 \in C^\ell (\mathbb {R}^s)$ satisfies

34 $$\begin{aligned} \alpha \ge \min \left\{ -\log _m \rho _{\mathcal A}, -\limsup _{k \rightarrow \infty } \frac{\log _m \delta _k}{k}\right\} . \end{aligned}$$

##### Proof

Let $k \ge 1$, $|\nu |=\ell$, and $h \in \mathbb {R}^s$ satisfy $m^{-(k+1)}< \Vert h\Vert _\infty \le m^{-k}$. To derive the estimate in (33), we use the triangle inequality

35 $$\begin{aligned} \Vert D^\nu \phi _1(\cdot +h)-D^\mu \phi _1(\cdot )\Vert _\infty\le & {} \Vert D^\mu \phi _1(\cdot +h)-D^\mu f_k(\cdot +h) \Vert _\infty + \Vert D^\mu \phi _1-D^\mu f_k\Vert _\infty \nonumber \\&+ \Vert D^\mu f_k(\cdot +h)-D^\mu f_k(\cdot )\Vert _\infty , \end{aligned}$$

where $\{f_k, \ k \ge 1\}$ are defined in (30), and estimate each of the summands on the right hand side. Note that, for $\Delta _h f_k:=f_k(\cdot +h)-f_k(\cdot )$, we have

$$\begin{aligned} \Delta _h D^\mu f_k =m^{\ell k}{\mathcal T}^{(1)} \ldots {\mathcal T}^{(k)} \Delta _{m^k h} D^\mu f. \end{aligned}$$

Due to $\Vert m^k h\Vert _\infty \le 1$ and by the definition of $\Delta _h$, we have

$$\begin{aligned} \hbox {supp} \Delta _{m^k h}D^\nu f \subset \hbox {supp} f+[-1,1]^s, \end{aligned}$$

where without loss of generality we assume that $\left( \hbox {supp} f+[-1,1]^s \right) \cap \mathbb {Z}^s \subseteq K$. Define the vector-valued function

$$\begin{aligned} v(x):=(\Delta _{m^k h} D^\nu f(x+\alpha ))_{\alpha \in K}, \quad x \in [0,1]^s. \end{aligned}$$

By the same argument as in the proof of Proposition 2 and by the definition of the operator $\Delta _h$, the first ${\sum _{j=1}^{\ell +1}} d_j$ components of *v* are zero for all $x \in \mathbb {R}^s$. Therefore, by Lemma 3, we get

$$\begin{aligned} \Vert T_{\varepsilon _1}^{(1)} \ldots T_{\varepsilon _k}^{(k)} v(x)\Vert \le C_1 m^{-\ell k} \eta _k \Vert v(x)\Vert \le C_1 m^{-\ell k}\eta _k \, 2 \, C_2 \, |K|, \quad x \in \mathbb {R}^s, \end{aligned}$$

where $\Vert v(x)\Vert \le 2 \, C_2 \, |K|$, $x \in \mathbb {R}^s$, due to $\max _{|\nu |=\ell }\Vert D^\nu f\Vert _\infty \le C_2$. Thus,

$$\begin{aligned} \Vert D^\nu f_k(\cdot +h)-D^\nu f_k(\cdot )\Vert _\infty \le C_3 \eta _k, \quad C_3:=C_1 m^{-\ell k} \, 2 \, C_2 \, |K|. \end{aligned}$$

The estimates for the two remaining terms in (35) and, thus, the estimate (33) follow by (31). Next, we derive the lower bound for the Hölder exponent $\alpha$ of $\phi _1$. Note that, by definition of $\sigma _k$, we have the equivalence

$$\begin{aligned} \lim \sup _{k \rightarrow \infty } \sigma _k^{1/k} \ge 1 \Leftrightarrow \ell + \lim \sup _{k \rightarrow \infty } \frac{\log _m\delta _k}{k} \ge 0. \end{aligned}$$

Thus, if $\lim \sup _{k \rightarrow \infty } \sigma _k^{1/k} \ge 1$, then $\min \{-\log _m \rho _{\mathcal A}, -\limsup \frac{\log _m \delta _k}{k} \}\le \ell$ and the estimate (34) holds, since $\phi _1 \in C^\ell (\mathbb {R}^s)$ and, thus, $\alpha \ge \ell$. Otherwise, if $\lim \sup _{k \rightarrow \infty } \sigma _k^{1/k} < 1$, then there exists $\theta$ such that $\lim \sup _{k\rightarrow \infty } \sigma _k^{1/k}< \theta <1$ and, thus, a constant $C_0>0$ such that $\sigma _k \le C_0 \theta ^k$, $k \ge 1$. Therefore, by definition of $\eta _k$ and using the estimate (33), we get

$$\begin{aligned} \Vert \Delta _h D^\nu \phi _1 \Vert _\infty\le & {} C\left( \eta _k+\sum _{n=k+1}^{\infty } \eta _n \right) =C \left( \sum _{j=0}^k \sigma _j q^{k-j}+\frac{1}{1-q} \sum _{n=k+1}^\infty \sigma _n \right) \\ {}\le & {} C \, C_0\left( \sum _{j=0}^k \theta ^j q^{k-j}+\frac{1}{1-q}\sum _{n=k+1}^\infty \theta ^n \right) \\\le & {} C \, C_0\left( \sum _{j=0}^k \max \{\theta ,q\}^j \max \{\theta ,q\}^{k-j}+ \frac{\theta ^{k+1}}{1-q}\sum _{n=k+1}^\infty \theta ^{n-(k+1)} \right) \\\le & {} C \, C_0\left( (k+1) \max \{\theta ,q\}^k+ \frac{\theta ^{k+1}}{(1-q)(1-\theta )}\right) . \end{aligned}$$

Therefore, due to $0 \le 1-q<1$, we get

$$\begin{aligned} \Vert \Delta _h D^\nu \phi _1 \Vert _\infty \le C_4 (k+1) \max \{\theta ,q\}^k, \quad C_4:=\max \left\{ 1, \frac{\theta }{1-\theta } \right\} \, \frac{C\, C_0}{1-q}. \end{aligned}$$

Moreover, due to $\Vert h\Vert _\infty \le m^{-k}$, we have

$$\begin{aligned} \max \{\theta ,q\}^k=m^{k \, \text {log}_m \max \{\theta ,q \}} \le \Vert h\Vert _\infty ^{-\text {log}_m \max \{\theta ,q\}} \end{aligned}$$

and, from $\frac{1}{m}\Vert h\Vert _\infty \le m^{-(k+1)}$, we get $(k+1) \le \log _m \frac{m}{\Vert h\Vert _\infty }$. Thus,

$$\begin{aligned} \Vert \Delta _h D^\nu \phi _1\Vert _\infty \le C_1 \log _m \left( \frac{m}{\Vert h\Vert _\infty } \right) \, \Vert h\Vert _\infty ^{-\log _m \max \{\theta ,q\}}. \end{aligned}$$

Note that for any $\epsilon \in (0,1)$, due to the fact that $-\log (t)$ is bounded by $t^{-\epsilon }$ for sufficiently small *t*, we get, for small $\Vert h\Vert _\infty$,

$$\begin{aligned} \Vert \Delta _h D^\nu \phi _1\Vert _\infty \le C_1 \Vert h\Vert _\infty ^{-\log _m \max \{\theta ,q\}-\epsilon }. \end{aligned}$$

By (22) and (23), we have $q>m^\ell \rho _{\mathcal A}$. Thus, since $\theta >\lim \sup _{k\rightarrow \infty } \sigma _k^{1/k}$, we get

$$\begin{aligned} \alpha \ge \ell -\log _m \max \{\theta ,q\}= & {} \ell -\max \left\{ \ell +\log _m \rho _{\mathcal A}, \ell +\lim \sup _{k\rightarrow \infty } \frac{\log _m\delta _k}{k}\right\} \\ {}= & {} \min \left\{ -\log _m \rho _{\mathcal A}, -\lim \sup _{k\rightarrow \infty } \frac{\log _m\delta _k}{k}\right\} . \end{aligned}$$

$$\square $$

Combining Propositions 2 and 3, we complete the proof of Theorem 2.

### Rapidly vanishing approximate sum rules defects

The following immediate consequence of Theorem 2 states that, if the sequence of defects $\{\delta _k, \ k \ge 1\}$ of the approximate sum rules decays fast, then the lower bound on the Hölder exponent $\alpha$ of $\phi _1$ only depends on the joint spectral radius $\rho _{\mathcal A}$ of the set ${\mathcal T}_{\mathcal A}|_{V_\ell }$.

#### Corollary 3

Assume that the symbols of $\{ S_{{\mathbf a}^{(k)}}, \ k \ge 1\}$ satisfy approximate sum rules of order $\ell +1$ and $\rho ({\mathcal T}_{\mathcal A}|_{V_\ell })<m^{-\ell }$. If $\limsup \nolimits _{k \rightarrow \infty } \delta _k^{1/k} \, < \, \rho _{\mathcal A}$, then $\alpha \, \ge \, - \log _m \rho _{\mathcal A}$.

Next, in this subsection we prove Theorem 3 stated in the Introduction. It shows that the inequality $\alpha \, \ge \, - \log _m \rho _{\mathcal A}$ in Corollary 3 becomes equality, if the set ${\mathcal A}$ of the limit points of the sequence $\{\mathbf {a}^{(k)}, \ k\ge 1 \}$ consists only of a single element $\mathbf {a}$ and the corresponding refinable limit function of $S_\mathbf {a}$ is stable. Note that Theorem 3 is a generalization of a well-known fact about the exact Hölder regularity of stationary schemes in the stable case.

In the proof of Theorem 3 we make use of several auxiliary facts on long matrix products. The first one of them is stated in the following lemma which is a special case of [59, Proposition 2].

#### Lemma 4

Let $\mathcal {M}$ be a compact set of $d\times d$ matrices and $y \in \mathbb {R}^d$. If $\rho (\mathcal M) > 1$ and *y* does not belong to a common invariant subspace of the matrices in $\mathcal {M}$, then the sequence $\left\{ \max _{P_n \in {\mathcal M}^n} \Vert P_n y \Vert , \ n \ge 1 \right\}$ diverges as $n \rightarrow \infty$.

Lemma 4 and the definition of the sequence $\left\{ \max _{P_n \in {\mathcal M}^n} \Vert P_n y \Vert , \ n \ge 1 \right\}$ yield

#### Lemma 5

Let $\mathcal {M}$ be a compact set of $d\times d$ matrices and $y \in \mathbb {R}^d$. If $\rho (\mathcal M) > 1$ and *y* does not belong to a common invariant subspace of the matrices in $\mathcal M$, then for any $L \in \mathbb {N}$ there exists $n \ge L$ such that

$$\begin{aligned} \Vert M_1\ldots M_n y\Vert \,> \Vert y\Vert \ \quad \hbox {and} \quad \, \Vert M_1\ldots M_n y\Vert \, > \Vert M_{n-i}\ldots M_n y\Vert \, , \ i = 0, \ldots , n-2, \end{aligned}$$

for $$M_j \in {\mathcal M}$$.

#### Proof

Let $L \in \mathbb {N}$ and $C_L = \max \, \bigl \{\Vert P_j y\Vert \ \bigl | \ P_j \in {\mathcal M}^j, j \le L\, \bigr \}$. Then the shortest product $P_n \in {\mathcal M}^n$ such that $\Vert P_n y\Vert > C_L$ (the set of such products is nonempty by Lemma 4) possesses the desired property and has its length bigger than *m*.$\square$

Next, we adapt Lemma 5 to the non-stationary setting. The proof of the following result is similar to the proof of Lemma 5 and we omit it.

#### Lemma 6

Let $\mathcal {M}$ and $\mathcal M^{\, (k)}$, $k \ge 1$, be compact sets of $d\times d$ matrices and $y \in \mathbb {R}^d$. Assume that $\rho (\mathcal M) > 1$, the sequence $\{\mathcal M^{\, (k)}, \ k \ge 1\}$ converges to $\mathcal M$ and *y* does not belong to a common invariant subspace of the matrices in $\mathcal M$. Then there exists $L \in \mathbb {N}$ and $C>0$ such that for any $\tilde{L} \ge L$ there exists $n \ge \tilde{L}$ such that, for $M_j \in {\mathcal M}^{(j+L-1)}$,

$$\begin{aligned}&\, \Vert M_1\ldots M_n y\Vert \,> C\Vert y\Vert \ \quad \hbox {and} \quad \, \Vert M_1\ldots M_n y\Vert \, > C \Vert M_{n-i}\ldots M_n y\Vert \, ,\\&\quad \ i = 0, \ldots , n-2. \end{aligned}$$

We are ready to prove Theorem 3.

#### Proof of Theorem 3

Due to Corollary 3, we only need to show that $\alpha \le -\log _m \rho _{\mathbf a}$. Furthermore, by Lemma 1, it suffices to show that $\alpha =\alpha _{\phi _n} \le -\log _m \rho _{\mathbf a}$ for some $n \ge 1$. We choose an appropriate *n* in the following way. Firstly, *n* should be such that

$$\begin{aligned} \rho \left( \{Q_\varepsilon ^{(k)}, \ \varepsilon \in E, \ k \ge n\}\right) < \rho _{\mathbf a}. \end{aligned}$$

(see Sect. 3.1 for the definition of the matrices $Q_\varepsilon ^{(k)}$). Secondly, since by assumption, there exists $\beta >0$ such that

$$\begin{aligned} \displaystyle {\limsup _{k \rightarrow \infty } \delta _k^{1/k}}< \beta < \rho _\mathbf {a}, \end{aligned}$$

thus, we can choose *n* such that for any constant $C_0>0$ we have $\delta _k < C_0 \beta ^k$ for $k \ge n$. At the end of the proof we specify the particular constant $C_0$ needed for our argument. Next, define

$$\begin{aligned} v(x)=\left( D^\nu \phi _n(x+\alpha ) \right) _{\alpha \in K}, \quad x \in [0,1]^s, \quad \nu \in \mathbb {N}_0^s, \quad |\nu |=\ell . \end{aligned}$$

Let $k \ge 1$. By definition of $\phi _n$, for $x=\sum _{j=1}^k \varepsilon _j m^{-j}$, $\varepsilon _j \in E$, and $\Vert h\Vert _\infty \le m^{-1}$, we have

36 $$\begin{aligned} \Delta _{m^{-k}h} v(x)= m^{\ell } T^{(n)}_{\varepsilon _1} \Delta _{m^{-k+1}h} v\left( \sum _{j=2}^k \varepsilon _j m^{-j}\right) =m^{\ell k} T^{(n)}_{\varepsilon _1} \ldots T^{(n+k-1)}_{\varepsilon _k} \Delta _{h} v(0). \end{aligned}$$

By the same argument as in Proposition 2, the first $L=\sum _{j=1}^{\ell +1} d_j$ components of the vector $y:=\Delta _{h} v(0)$ are zero. Denote by $\tilde{y}:=(y_{L+1}, \ldots , y_{|K|})^T$ the non-zero components of *y*. W.l.o.g. we can assume that the vector $\tilde{y}$ does not belong to any common invariant subspace of the matrices in $\{T_{\varepsilon ,\mathbf {a}}|_{V_\ell } \ : \ \varepsilon \in E\}$. Otherwise, due to the stability of $\phi$ we have

$$\begin{aligned} \alpha _\phi =-\log _m \rho _\mathbf {a}=-\log _m \rho \{ T_{\varepsilon ,\mathbf {a}}|_{W} \ : \ \varepsilon \in E\}, \end{aligned}$$

where *W* is the smallest subspace of $V_\ell$ such that it is invariant under all operators in $\{ T_{\varepsilon ,\mathbf {a}} \ : \ \varepsilon \in E\}$ and such that $T_{\varepsilon ,\mathbf {a}}|_{W}$, $\varepsilon \in E$, do not have any common invariant subspace. For simplicity, we assume that $W=V_\ell$, but the same argument we give below would apply, if *W* is a proper subspace of $V_\ell$. Let $r \in (\beta , \rho _\mathbf {a})$ be a real number. The sets

$$\begin{aligned} {\mathcal M}=\{r^{-1}T_{\varepsilon ,\mathbf {a}}|_{V_\ell }, \ \varepsilon \in E\} \quad \hbox {and} \quad {\mathcal M}^{(k)}=\{r^{-1}Q^{(n+k-1)}_{\varepsilon }, \ \varepsilon \in E\}, \quad k\ge 1, \end{aligned}$$

and the vector $\tilde{y}$ satisfy the assumptions of Lemma 6. Thus, we can appropriately modify *n* chosen above to get

37 $$\begin{aligned}&\Vert Q^{(n)}_{\varepsilon _1} \ldots Q^{(n+k-1)}_{\varepsilon _k} \tilde{y}\Vert \,> C r^k \Vert \tilde{y}\Vert \ \quad \hbox {and} \nonumber \\&\Vert Q^{(n)}_{\varepsilon _1} \ldots Q^{(n+k-1)}_{\varepsilon _k} \tilde{y}\Vert \, > C r^{k-i}\Vert Q^{(n+k-i)}_{\varepsilon _{k-i+1}} \ldots Q^{(n+k-1)}_{\varepsilon _k} \tilde{y}\Vert ,\quad i = 1, \ldots , k-1.\quad \quad \quad \end{aligned}$$

Denote by $H^{(n+k-i)}_j\in \mathbb {R}^{ 1 \times |K|}$ the *j*th row of the matrix $T^{(n+k-i)}_{\varepsilon }$, $\varepsilon \in E$. Define $y_0:=y$, the we have

38 $$\begin{aligned} T^{(n+k-1)}_{\varepsilon _k} y_0 \ = \left( \begin{array}{c} 0\\ \vdots \\ 0 \\ Q^{(n+k-1)}_{\varepsilon _{k}} \tilde{y}\end{array} \right) + \ \sum _{j=1}^{L} \langle H^{(n+k-1)}_j, y_0\rangle e_j\, , \end{aligned}$$

where $e_j$, $j=1, \ldots , L$, are the standard first *L* unit vectors of $\mathbb {R}^{|K|}$ and $\langle H^{(n+k-1)}_j, y_0\rangle$ is the scalar product of the vectors $H^{(n+k-1)}_j$ and $y_0$. Define $$y_1:=\left( \begin{array}{cccc} 0&\dots&0&Q^{(n+k-1)}_{\varepsilon _{k}} \tilde{y} \end{array} \right) ^T$$. Then, applying $T^{(n)}_{\varepsilon _1} \ldots T^{(n+k-2)}_{\varepsilon _{k-1}}$ to both sides of (38), we get

$$\begin{aligned} T^{(n)}_{\varepsilon _1} \ldots T^{(n+k-2)}_{\varepsilon _{k-1}} y_1= T^{(n)}_{\varepsilon _1} \ldots T^{(n+k-1)}_{\varepsilon _k} y_0 - \sum _{j=1}^{L} \langle H^{(n+k-1)}_j, y_0\rangle \, T^{(n)}_{\varepsilon _1} \ldots T^{(n+k-2)}_{\varepsilon _{k-1}} e_j, \end{aligned}$$

and, thus, by triangle inequality,

$$\begin{aligned} \Vert T^{(n)}_{\varepsilon _1} \ldots T^{(n+k-1)}_{\varepsilon _k} y_0 \Vert\ge & {} \Vert T^{(n)}_{\varepsilon _1} \ldots T^{(n+k-2)}_{\varepsilon _{k-1}} y_1\Vert \\&- \ \sum _{j=1}^{L}| \langle H^{(n+k-1)}_j, y_0\rangle |\, \Vert T^{(n)}_{\varepsilon _1} \ldots T^{(n+k-2)}_{\varepsilon _{k-1}} e_j\Vert . \end{aligned}$$

Note that *n* is such that, for any $n+k-i \ge n$, the matrix $T^{(n+k-i)}_{\varepsilon }$ is bounded by the matrix $R_{n+k-i}$, in the sense of Lemma 3. Then, due to the structure of $y_0$, we have $| \langle H^{(n+k-1)}_j, y_0\rangle | = \mathcal{O}( m^{-(\ell -j+1)(n+k-1)}\sigma _{n+k-1}) \Vert y_0 \Vert$. By Lemma 3, we also obtain the estimate $\Vert T^{(n)}_{\varepsilon _1} \ldots T^{(n+k-2)}_{\varepsilon _{k-1}} e_j \Vert = \mathcal{O}(m^{-(j-1)k})$, $j=1, \ldots , L$. And, thus,

$$\begin{aligned}&\sum _{j=1}^{L}| \langle H^{(n+k-1)}_j, y_0\rangle |\, \Vert T^{(n)}_{\varepsilon _1} \ldots T^{(n+k-2)}_{\varepsilon _{k-1}} e_j\Vert \\&\quad = \sum _{j=1}^{L} \mathcal{O}( m^{-(\ell -j+1)(n+k-1)} \sigma _{n+k-1} m^{-(j-1)k}) \Vert y_0\Vert \\&\quad = \mathcal{O}( m^{-\ell (n+k-1)} \sigma _{n+k-1}) \Vert y_0\Vert . \end{aligned}$$

The definition of $\sigma _k$ and the choice of $\beta$ yield $\mathcal{O}( m^{-\ell (n+k-1)} \sigma _{n+k-1})=\mathcal{O}( \delta _{n+k-1}) < \tilde{C} C_0 \beta ^{n+k-1}$, $\tilde{C}>0$. Therefore,

$$\begin{aligned} \Vert T^{(n)}_{\varepsilon _1} \ldots T^{(n+k-1)}_{\varepsilon _k} y_0 \Vert \ge \Vert T^{(n)}_{\varepsilon _1} \ldots T^{(n+k-2)}_{\varepsilon _{k-1}} y_1\Vert - \tilde{C} C_0 \beta ^{n+k-1} \Vert y_0\Vert . \end{aligned}$$

Set $$y_i:=\left( \begin{array}{cccc} 0&\ldots&0&Q^{(n+k-i)}_{\varepsilon _{k-i+1}} \ldots Q^{(n+k-1)}_{\varepsilon _{k}} \tilde{y} \end{array} \right) ^T$$, $i=2, \ldots , k$. Then, analogous successive argument for $\Vert T^{(n)}_{\varepsilon _1} \ldots T^{(n+k-i)}_{\varepsilon _{k-i+1}} y_{i-1}\Vert$, $i=2, \ldots ,k$, yields

$$\begin{aligned} \Vert T^{(n)}_{\varepsilon _1} \ldots T^{(n+k-1)}_{\varepsilon _k} y_0 \Vert \ge \Vert y_k\Vert - \tilde{C} C_0 \sum _{i=0}^{k-1} \beta ^{n+k-i-1} \Vert y_i\Vert . \end{aligned}$$

From (37) we get $\Vert y_i\Vert <r^{-k+i}C^{-1}\Vert y_k\Vert$, $i=0, \ldots , k-1$, which implies

$$\begin{aligned} \Vert T^{(n)}_{\varepsilon _1} \ldots T^{(n+k-1)}_{\varepsilon _k} y_0 \Vert> & {} \left( 1-\frac{\tilde{C} C_0 \beta ^{n-1}}{C} \sum _{i=0}^{k-1} \left( \frac{\beta }{r}\right) ^{k-i} \right) \Vert y_k\Vert \\> & {} \left( 1-\frac{\tilde{C} C_0 \beta ^{n-1}}{C (1-\frac{\beta }{r})} \right) \Vert y_k\Vert . \end{aligned}$$

In the second estimate above we used the fact that $\beta <r$. Choose $0<C_0<\frac{C(1-\frac{\beta }{r})}{\tilde{C} \beta ^{n-1}}$ and define $C_1:=1-\frac{\tilde{C} C_0 \beta ^{n-1}}{C (1-\frac{\beta }{r})}>0$. Therefore, by (37), we have $\Vert y_k\Vert >C r^k \Vert y_0\Vert$ and, thus,

$$\begin{aligned} \Vert T^{(n)}_{\varepsilon _1} \ldots T^{(n+k-1)}_{\varepsilon _k} y_0 \Vert> C_1 \Vert y_k\Vert > C_1 C \, r^k \Vert y_0\Vert , \quad k \ge 1. \end{aligned}$$

Finally, this estimate and (36) yield $\Vert \Delta _{m^{-k}h}v(x)\Vert > C_1 C r^k m^{\ell k} \Vert y_0\Vert$, $k \ge 1$. Therefore, the Hölder exponents of all $D^\nu \phi _n$, $\nu \in \mathbb {N}_0^s$, $|\nu |=\ell$, are bounded from above by $-\ell -\log _m r$ and, thus, $\alpha =\alpha _{\phi _n} \le -\log _m r$. Taking the limit as *r* goes to $\rho _{\mathbf a}$, we obtain the desired estimate $\alpha \le -\log _m \rho _{\mathbf a}$.$\square$

If the symbols of the scheme $\{ S_{{\mathbf a}^{(k)}}, \ k \ge 1\}$ satisfy sum rules of order $\ell +1$, then we get the following immediate consequence of Theorem 3.

#### Corollary 4

Let $\ell \ge 0$. Assume the stationary scheme $S_{\mathbf {a}}$ is $C^\ell$-convergent with the stable refinable basic limit function $\phi$ whose Hölder exponent $\alpha _\phi$ is $\ell \le \alpha _\phi <\ell +1$. If the symbols of the scheme $\{ S_{{\mathbf a}^{(k)}}, \ k \ge 1\}$ satisfy sum rules of order $\ell +1$ and $\lim _{k \rightarrow \infty } \mathbf {a}^{(k)}=\mathbf {a}$, then $\{ S_{{\mathbf a}^{(k)}}, \ k \ge 1\}$ is $C^\ell$-convergent and the Hölder exponent of its limit functions is also $\alpha _\phi$.

### Applications and examples

In the this section, see Sect. 3.6.1, we prove the conjecture formulated in [32], which stipulates the Hölder regularity of the generalized Daubechies wavelets. The proof of this conjecture is a direct consequence of Theorem 3. We also determine the exact Hölder regularity of some of such generalized Daubechies wavelets. Moreover, in Sect. 3.6.2, we illustrate our theoretical convergence and Hölder regularity results with several deliberately simple examples for which though neither the results of [18] nor the ones in [34, 36] are applicable.

Note that, in this section, we use the techniques from [41] that allow for exact computation of the joint spectral radius of the corresponding matrix sets. The method in [41] determines the so-called *spectrum maximizing product* of such sets, which yields the exact value of the joint spectral radius.

#### Definition 13

Let ${\mathcal M}$ be a compact collection of square matrices. The product $P:=M_1 \ldots M_m$, $M_j \in {\mathcal M}$, is spectrum maximizing, if $\rho ({\mathcal M})=\rho (P)^{1/m}$, where $\rho (P)$ is the spectral radius of *P*.

#### Exact Hölder regularity of generalized Daubechies wavelets

The non-stationary Daubechies wavelets are defined and studied in [32] and are obtained from Daubechies wavelets in [25] by suitable perturbation of the roots of the stationary symbols. Let $n \ge 2$. To an arbitrary set $\Lambda _n: = \{\lambda _0, \ldots , \lambda _{n-1}\}$ of real numbers $\lambda _j$, $j=0, \ldots , n-1$, the authors in [32] associate the generalized Daubechies wavelet function $\psi ^{\Lambda _n}$. The corresponding refinable function

$$\begin{aligned} \phi ^{\Lambda _n}:=\lim _{k \rightarrow \infty } S_{\mathbf {a}^{(k)}} S_{\mathbf {a}^{(k-1)}} \ldots S_{\mathbf {a}^{(1)}} {\varvec{\delta }}\end{aligned}$$

is the limit function of a non-stationary subdivision scheme $\{S_{\mathbf {a}^{(k)}}, \ k \ge 1\}$ reproducing exponential polynomials, i.e., solutions of the ODE of order *n* with constant coefficients and with spectrum $\Lambda _n$. The interested reader can find more details on the construction and properties of these wavelets $\psi ^{\Lambda _n}$, $n \ge 2$, in [32].

Next we would like to mention the following two properties of these masks $\{\mathbf {a}^{(k)}$, $k \ge 1\}$:

(i) the sequence of masks $\{ \mathbf {a}^{(k)}, \ k \ge 1\}$ converges to the mask $\mathbf {m}_n$ of the classical *n*th Daubechies refinable function $\varphi _n:=\lim _{k \rightarrow \infty } S_{\mathbf {m}_n}^k {\varvec{\delta }}\, ;$
(ii) the corresponding symbols $\{ a_*^{(k)}(z), \ k \ge 1\}$ satisfy approximate sum rules of order *n* with $\delta _k = \mathcal{O}(2^{-nk}), \, k \ge 1$.

In [32] the authors estimated the Hölder exponent of the generalized Daubechies wavelets and conjectured that it equals to the Hölder exponent of the usual (stationary) Daubechies wavelets (Conjecture 1 stated in Sect. 1.2). The following result proves this conjecture.

##### Theorem 5

Let $n\ge 2$. For every set $\Lambda _n = \{\lambda _0, \ldots , \lambda _{n-1}\}$, the Hölder regularity of the generalized Daubechies type wavelet $\psi ^{\Lambda _n}$ is equal to the Hölder regularity of the classical Daubechies wavelet $\psi _n$ derived from $\varphi _n$.

##### Proof

We invoke Theorem 3. Since a compactly supported wavelet function has the same regularity as the corresponding refinable function, we need to show that the functions $\phi ^{\Lambda _n}$ and $\varphi _{n}$ have the same regularity. The non-stationary subdivision scheme $\{S_{\mathbf {a}^{(k)}}, \ k \ge 1\}$ generating $\phi ^{\Lambda _n}$ satisfies the assumptions of Theorem 2 with $\ell =n-1$ and ${\mathcal A}=\{\mathbf {m}_n\}$. Indeed, the masks of the scheme $\{S_{\mathbf {a}^{(k)}}, \ k \ge 1\}$ are constructed in [32] in such a way that they converge to the mask $\mathbf {m}_n$. The Daubechies refinable function $\varphi _n$ is stable and, hence, its Hölder exponent is $\alpha _{\varphi _n} = - \log _2 \rho _{\mathcal A}$. It is well-known that $\alpha _{\varphi _n} < n$, therefore $\rho _{\mathcal A}> 2^{-n}$. Thus, by (*ii*) we have $\limsup _{k \rightarrow \infty } \delta _k^{1/k} \le 2^{-n} < \rho _{\mathcal A}$. Therefore, all assumptions of Theorem 3 are satisfied and the Hölder exponent $\alpha$ of $\phi ^{\Lambda _n}$ satisfies $\alpha = \alpha _{\varphi _n}=- \log _2 \rho _{{\mathcal A}}$.$\square$

In [32] the Hölder exponent $\alpha$ is estimated by the rate of decay of the Fourier transform $\hat{\phi }^{\Lambda _n}$ of $\phi ^{\Lambda _n}$. It is well-known that for any continuous, compactly supported function *f*, its Hölder exponent $\alpha _f$ satisfies

$$\begin{aligned} \eta (f) -1 \le \alpha _f \le \eta (f), \quad \eta (f) = \sup \, \bigl \{\beta \ge 0 \ : \ |{\widehat{f}}(\omega )| \le C(1 + |\omega |)^{-\beta } \, , \ \omega \in \mathbb {R}\, \bigr \}, \end{aligned}$$

and this gap of length 1 is, in general, unavoidable [68]. In [32, Theorem 29] the authors show that $\eta (\phi ^{\Lambda _n}) \ge \eta (\varphi _{n})$, which, thus, implies the following lower bound for the Hölder exponent $\alpha$ of $\phi ^{\Lambda _n}$ $\alpha \, \ge \, \eta (\varphi _{n}) -1.$ Using lower bounds for the values $\eta (\varphi _{n})$ known from the literature, one can estimate the regularity of the generalized Daubechies wavelets. Table in (39) compares those rough bounds given in [25] (computed by the method of invariant cycles) with the exact values of $\alpha = -\log _2 \rho _{\mathcal A}$, which we compute using the techniques in [41].39


#### Further examples

In this subsection we apply our convergent and regularity results to several deliberately simple non-stationary subdivision schemes whose analysis was impossible so far. These examples are constructed only for illustration purposes.

##### Example 2

We start with a non-stationary subdivision scheme with a general dilation matrix *M* and masks which are level dependent convex combination of two multivariate masks $\mathbf {a},\mathbf {b} \in \ell _0(\mathbb {Z}^s)$. We assume that $\mathbf {a}$ defines a (stationary) convergent subdivision scheme and that $\mathbf {b}$ satisfies sum rules of order 1. Convex combinations of such subdivision masks were also investigated in [9, 17]. In particular, we define the non-stationary subdivision scheme $\{S_{\mathbf {a}^{(k)}}, \ k \ge 1 \}$ by

40 $$\begin{aligned} \mathbf {a}^{(k)}:=\left( 1-\frac{1}{k}\right) \mathbf {a}+\frac{1}{k} \mathbf {b}, \quad k \ge 1. \end{aligned}$$

This non-stationary scheme does not satisfy the condition in (2) for $\ell =0$, since $|\mathrm{a}^{(k)}(\alpha )-\mathrm{a}(\alpha )| = |\mathrm{b}(\alpha )-\mathrm{a}(\alpha )| \frac{1}{k}$,

$$\begin{aligned} \sum _{k \in \mathbb {N}}\max _{\varepsilon \in E} \left\{ \sum _{\alpha \in \mathbb {Z}^s} |\mathrm{a}^{(k)}(\varepsilon +M\alpha )-\mathrm{a}(\varepsilon +M\alpha )| \right\} \nless \infty \end{aligned}$$

Nevertheless, $\{S_{\mathbf {a}^{(k)}}, \ k \ge 1 \}$ satisfies the assumptions of Theorem 4, since, by construction, all symbols satisfy approximate sum rules of order 1 and $\lim _{k \rightarrow \infty } \mathbf {a}^{(k)}=\mathbf {a}$. Therefore, we are able to conclude that the scheme is at least $C^0$-convergent. Moreover, in the case $M=mI$, the assumptions that $S_\mathbf {a}$ is $C^\ell$-convergent and that $\mathbf {b}$ satisfies sum rules of order $\ell +1$, imply, by Theorem 2, that the Hölder regularity of the scheme in (40) is at least as high as for $S_\mathbf {a}$. Indeed, for $s=2$ and $M=2I$, let $\mathbf {a}$ be the mask of the butterfly scheme ([38] with $\omega =1/16$) and $\mathbf {b}$ be the mask of the Courant element, the box spline $B_{111}$. Then, using the method in [41], we compute $\rho ({\mathcal T}_{\mathbf {a}}|_{V_1})=1/4$ and, thus, the scheme $\{S_{\mathbf {a}^{(k)}}, \ k \ge 1 \}$ is $C^1$-convergent and its Hölder exponent is $\alpha = 2$.

In the next example we construct non-stationary schemes with sets of limit points ${\mathcal A}$ of cardinality 2.

##### Example 3

Let $\mathcal I\subset \mathbb {N}$ be some infinite set, such that $\mathbb {N}{\setminus } \mathcal{I}$ is also infinite. We consider the non-stationary scheme with the masks

41 $$\begin{aligned} \mathbf {a}^{(k)}:=\left\{ \begin{array}{ll} \mathbf {a},\ &{}\quad k\in \mathcal{I}, \\ {\varvec{c}},\ &{}\quad k\in \mathbb {N}{\setminus } \mathcal{I}, \end{array} \right. \quad k \ge 1. \end{aligned}$$

We assume that the masks $\mathbf {a},\ {\varvec{c}}\in \ell _0(\mathbb {Z}^s)$ define stationary convergent subdivision schemes with the same dilation matrix *M*. Moreover, we assume that $\rho \left( {\mathcal T}_{\mathcal A}|_{V_0}\right) <1$, ${\mathcal A}=\{\mathbf {a}, \mathbf {c}\}$. Here the notion of asymptotic equivalence is not applicable, but Theorem 4 allows us to establish $C^0$-convergence of the scheme in (41). If $M=mI$ and $\mathbf {a},\ {\varvec{c}}$ are such that $\rho \left( {\mathcal T}_{\mathcal A}|_{V_\ell }\right) <m^{-\ell }$, Theorem 2 also yields a lower bound for the Hölder regularity of $\{S_{\mathbf {a}^{(k)}}, \ k \ge 1\}$.

For example, for $s=1$ and $M=2$, let

$$\begin{aligned} a_*(z):=\frac{1}{8}(1+z)^4 \quad \text{ and } \quad c_*(z):=\frac{1}{16}(-1+9z^2+16z^3+9z^4-z^6), \quad z \in \mathbb {C}{\setminus } 0, \end{aligned}$$

be the symbols of the cubic B-spline and the 4-point scheme ([31] with $\omega =1/16$), respectively. Using the method in [41] we obtain $\rho ({\mathcal T}_{{\mathcal A}}|_{V_1})=0.35385\ldots$. This tells us that the corresponding scheme $\{S_{\mathbf {a}^{(k)}}, \ k \ge 1\}$ has the Hölder exponent $\alpha \ge 1.49876\ldots$. For the computation of $\rho ({\mathcal T}_{{\mathcal A}}|_{V_1})$, we used the set ${\mathcal T}_{{\mathcal A}}|_{V_1}=\{T_1:=T_{0,\mathbf {c}}|_{V_1}, T_2 :=T_{1,\mathbf {c}}|_{V_1},T_3 :=T_{0,\mathbf {a}}|_{V_1},T_4:=T_{1,\mathbf {a}}|_{V_1} \}$ with

$$\begin{aligned}&\qquad T_1= \left( \begin{array}{rrrr} \frac{1}{8} &{} \frac{1}{8} &{} 0 &{} 0 \\ -\frac{1}{16} &{} \frac{3}{8} &{} -\frac{1}{16} &{} 0 \\ 0 &{} \frac{1}{8} &{} \frac{1}{8} &{} 0 \\ 0 &{} -\frac{1}{16} &{} \frac{3}{8} &{} -\frac{1}{16} \end{array} \right) , \qquad T_2= \left( \begin{array}{rrrr} -\frac{1}{16} &{} \frac{3}{8} &{} -\frac{1}{16} &{} 0 \\ 0 &{} \frac{1}{8} &{} \frac{1}{8} &{} 0 \\ 0 &{} -\frac{1}{16} &{} \frac{3}{8} &{} -\frac{1}{16} \\ 0 &{} 0 &{} \frac{1}{8} &{} \frac{1}{8} \end{array} \right) , \\&\qquad T_3= \left( \begin{array}{rrrr} \frac{1}{8} &{} 0 &{} 0 &{} 0 \\ \frac{1}{8} &{} \frac{1}{4} &{} \frac{1}{8} &{} 0 \\ 0 &{} 0 &{} \frac{1}{8} &{} \frac{1}{4} \\ 0 &{} 0 &{} 0 &{} 0 \end{array} \right) , \qquad T_4= \left( \begin{array}{rrrr} \frac{1}{4} &{} \frac{1}{8} &{} 0 &{} 0 \\ 0 &{} \frac{1}{8} &{} \frac{1}{4} &{} \frac{1}{8} \\ 0 &{} 0 &{} 0 &{} \frac{1}{8} \\ 0 &{} 0 &{} 0 &{} 0 \end{array} \right) . \end{aligned}$$

The spectrum maximizing product we obtain is $T_1 (T_1 T_3)^{13}$. If, instead of the mask $\mathbf {a}$ above, we take the mask of the quadratic B-spline, then we obtain $\rho ({\mathcal T}_{{\mathcal A}}|_{V_1})=0.35045\ldots$, which tells us that the corresponding scheme $\{S_{\mathbf {a}^{(k)}}, \ k \ge 1\}$ has Hölder exponent $\alpha \ge 1.51271\ldots$. For the computation of $\rho ({\mathcal T}_{{\mathcal A}}|_{V_1})$ we used the set ${\mathcal T}_{{\mathcal A}}|_{V_1}=\{T_1, T_2, T_5,T_6\}$ with

$$\begin{aligned}&T_5: = T_{0,\mathbf {a}}|_{V_1}=\left( \begin{array}{rrrr} \frac{1}{4} &{} 0 &{} 0 &{} 0 \\ 0 &{} \frac{1}{4} &{} \frac{1}{4} &{} 0 \\ 0 &{} 0 &{} 0 &{} \frac{1}{4} \\ 0 &{} 0 &{} 0 &{} 0 \\ \end{array} \right) , \qquad T_6 := T_{1,\mathbf {a}}|_{V_1}=\left( \begin{array}{rrrr} \frac{1}{4} &{} \frac{1}{4} &{} 0 &{} 0 \\ 0 &{} 0 &{} \frac{1}{4} &{} \frac{1}{4} \\ 0 &{} 0 &{} 0 &{} 0 \\ 0 &{} 0 &{} 0 &{} 0 \end{array} \right) . \end{aligned}$$

The spectrum maximizing product is $T_1 (T_1 T_5)^{2}$. Note that $\rho ({\mathcal T}_{{\mathcal A}}|_{V_1})$ can be bigger than either $\rho ({\mathcal T}_{\mathbf {a}}|_{V_1})$ or $\rho ({\mathcal T}_{\mathbf {c}}|_{V_1})$. It is also of interest that $\rho ({\mathcal T}_{{\mathcal A}}|_{V_1})$ decreases, if we replace the mask of the cubic B-spline by the mask of the less regular scheme corresponding to the quadratic B-spline.

The next example studies a univariate ternary ($M=3$) non-stationary scheme.

##### Example 4

We consider the alternating sequence of symbols

42 $$\begin{aligned} a_*^{(k)}(z):=\left\{ \begin{array}{ll} c_*^{(k)}(z):=z^{-6} K_1^{(k)} \ (z^2 + z + 1)^2 (z + 1) {\tilde{c}}_*^{(k)}(z), &{}\quad k\ \ \hbox {even,} \\ d_*^{(k)}(z):= z^{-6} K_2^{(k)} \ (z^2 + z + 1)^2 (z + 1) {\tilde{d}}_*^{(k)}(z), &{}\quad k\ \ \hbox {odd,} \end{array} \right. \quad k\ge 1, \end{aligned}$$

where $K_1^{(k)}$ and $K_2^{(k)}$ are suitable normalization constants and the factors ${\tilde{c}}_*^{(k)}(z)$ and ${\tilde{d}}_*^{(k)}(z)$ are defined byand

$$\begin{aligned} {\tilde{d}}_*^{(k)}(z):= \Big (z^2 + z + 1 \Big ) \Big ( w^{(k)}z^4 + 2w^{(k)}z^3 + (4(w^{(k)})^2-1)z^2 +2w^{(k)}z + 1 \Big ), \end{aligned}$$

with $$w^{(k)}:=\frac{1}{2}\left( e^{3^{-(k+1)}\lambda /2}+e^{-3^{-(k+1)}\lambda /2}\right) $$ and $$\lambda \in \mathbb {R}^+\cup i\mathbb {R}^+$$.

The corresponding non-stationary subdivision scheme $\{S_{{\mathbf {c}}^{(k)}},\ k\ge 1\}$ was considered in [10, 21]. In [21], the authors investigate the convergence of the sequence of symbols $\{{c}_*^{(k)}(z),\ k\ge 1\}$ to the symbol

$$\begin{aligned} {c}_*(z)=-z^{-6}\frac{1}{1296}(z^2 + z + 1)^4(z + 1)(35z^2 - 94z +35) \end{aligned}$$

of the ternary dual stationary 4-point Dubuc-Deslaurier scheme, which is known to be at least $C^2$-convergent. The sequence of the symbols of the non-stationary subdivision scheme $\{S_{{\mathbf {d}}^{(k)}},\ k\ge 1\}$ converges to the symbol

$$\begin{aligned} {d}_*(z)=-z^{-6}\frac{1}{162}(z^2 + z + 1)^5(z + 1), \end{aligned}$$

see [10]. The stationary scheme $S_\mathbf {d}$ is known to be at least $C^2$-convergent. We would like to remark that $\{S_{{ \mathbf {c}}^{(k)}},\ k\ge 1\}$ and $\{S_{{ \mathbf {d}}^{(k)}},\ k\ge 1\}$ are both schemes generating/reproducing certain spaces of exponential polynomials, see [20].

Using Theorem 2 with ${\mathcal A}=\{{\varvec{c}}, {\mathbf {d}}\}$ and the method in [41], we determine a lower bound for the Hölder regularity of the scheme $\{S_{\mathbf {a}^{(k)}}, \ k \ge 1\}$. Since we get $\rho ({{\mathcal T}}_{{\mathcal A}}|_{V_2})=0.04958\ldots$, the corresponding Hölder exponent satisfies $\alpha \ge 2.73437\ldots$. For the computation of $\rho ({{\mathcal T}}_{{\mathcal A}}|_{V_2})$ we use the set ${\mathcal T}_{{\mathcal A}}=\{T_1,T_2,T_3,T_4,T_5,T_6\}$ with $T_j:=1296\,T_{j-1,\mathbf {c}}|_{V_2}$, $j=1,2,3$,

$$\begin{aligned}&T_1=\left( \begin{array}{rrrr} 35 &{} 0 &{} 0 \\ -83 &{} -83 &{} -24 \\ 0 &{} 35 &{} -24 \\ \end{array} \right) ,\qquad \ T_2=\left( \begin{array}{rrrr} -24 &{} 35 &{} 0 \\ -24 &{} -83 &{} -83 \\ 0&{} 0 &{} 35 \\ \end{array} \right) ,\\&\qquad T_3=\left( \begin{array}{rrrr} -83&{}-24 &{} 35 \\ 35&{} -24 &{} -83 \\ 0&{} 0&{} 0 \end{array} \right) , \end{aligned}$$

and with $$T_j:=T_{j-4,\mathbf {d}}|_{V_2}$$, $$j=4,5,6$$,

$$\begin{aligned} T_4=\frac{1}{162}\left( \begin{array}{rrrr} 1 &{} 0 &{} 0 \\ 5 &{} 5 &{} 3 \\ 0 &{} 1 &{} 3 \end{array} \right) ,\qquad \quad T_5=\frac{1}{162}\left( \begin{array}{rrrr} 3 &{} 1 &{} 0 \\ 3 &{} 5 &{} 5 \\ 0&{} 0 &{} 0 \end{array} \right) , \qquad T_6=\frac{1}{162}\left( \begin{array}{rrrr} 5&{} 3 &{} 1 \\ 1&{} 3 &{} 5 \\ 0&{} 0&{} 0 \end{array} \right) . \end{aligned}$$

The spectrum maximizing product is $T_1 T_3$, which implies that the Hölder exponent $\alpha$ coincides with the Hölder exponent of the scheme $S_{\varvec{c}}$.

In the next two examples, we construct and analyze the regularity of a univariate and a multivariate non-stationary subdivision schemes obtained by suitable perturbations of the masks of the known stationary subdivision schemes. These non-stationary schemes are not asymptotically equivalent to any stationary scheme and, thus, the results of [34] are not applicable. Note though that these schemes satisfy approximate sum rules of order 2 and the other assumptions of Theorem 2.

##### Example 5

For $s=1$ and $M=2$, we consider the sequence of masks $\{\mathbf {a}^{(k)},\ k\ge 1\}$ with

43 $$\begin{aligned} \mathbf {a}^{(k)}:=\left\{ \left( \frac{1}{4} -\frac{1}{k}\right) ,\ \left( \frac{3}{4} -\frac{1}{k}+2^{-2k}\right) ,\ \left( \frac{3}{4} +\frac{1}{k}\right) ,\ \left( \frac{1}{4} +\frac{1}{k}+2^{-2k}\right) \right\} ,\quad k\ge 1. \end{aligned}$$

Obviously, $\lim _{k \rightarrow \infty } \mathbf {a}^{(k)}=\mathbf {a}$, where $\mathbf {a}=\left\{ \frac{1}{4},\ \frac{3}{4},\ \frac{3}{4},\, \frac{1}{4} \right\}$ is the mask of the Chaikin subdivision scheme [6]. It is easy to check that the symbols of this non-stationary scheme satisfy

$$\begin{aligned} a_*^{(k)}(1)-2=2^{-2k+1}, \quad a_*^{(k)}(-1)=-2^{-2k+1}\quad \hbox {and} \quad D a_*^{(k)}(-1)= 2^{-2k+2}, \quad k\ge 1, \end{aligned}$$

i.e. $\mu _k=\delta _k=2^{-2k+1}$ and, thus, the symbols satisfy approximate sum rules of order 2. To be able to apply Theorem 2, we need to rescale the masks $\mathbf {a}^{(k)}$ so that $\mu _k=0$, $k\ge 1$. It is easily done by multiplying each of the masks $\mathbf {a}^{(k)}$ by the factor $2/(2+\mu _k)$. After this modification the sequence $\{\delta _k, \ k \ge 1\}$ is still summable, since ${ \sum _{k \in \mathbb {N}} \frac{2 \delta _k}{2+\mu _k}< \sum _{k \in \mathbb {N}} \delta _k< \infty }.$ Hence, by Theorem 2 and the known fact that $\rho ({\mathcal T}_\mathbf {a}|_{V_1})=\frac{1}{4}$, the non-stationary scheme with masks in (43) is $C^1$-convergent with $\alpha = 2$.

##### Example 6

For $s=2$ and $M=2I$, we consider the sequence of masks $\{\mathbf {a}^{(k)},\ k\ge 1\}$ with for $k\ge 1$

44 $$\begin{aligned} \mathbf {a}^{(k)}=\frac{1}{16}\left( \begin{array}{ccccc} 0 &{} 2^{-2k} &{} 1+\frac{1}{k} &{} 2-\frac{1}{k} &{} 1-\frac{1}{k} \\ -\frac{1}{k} &{} 2-\frac{1}{k}+2^{-2k} &{} 6+\frac{1}{k}+2^{-2k} &{} 6+\frac{1}{k}+2^{-2k} &{} 2+2^{-2k} \\ 1-\frac{1}{k} &{} 6+\frac{1}{k} &{} 10+\frac{2}{k} &{} 6+\frac{1}{k} &{} 1-\frac{1}{k} \\ 2+ 2^{-k} &{} 6+\frac{1}{k}+2^{-2k} &{} 6+\frac{1}{k}+2^{-2k} &{} 2-\frac{1}{k}+2^{-k} &{} -\frac{1}{k} \\ 1-\frac{1}{k} &{} 2-\frac{1}{k} &{} 1+\frac{1}{k} &{} 2^{-2k} &{} 0 \\ \end{array} \right) \end{aligned}$$

Obviously, $\lim _{k \rightarrow \infty } \mathbf {a}^{(k)}=\mathbf {a}$, where $\mathbf {a}$ is the mask of the Loop subdivision scheme [52]. Note that the symbols of this non-stationary scheme satisfy approximate sum rules of order 2, since, we have $\mu _k=5\cdot 2^{-(2k+4)}$ and $\delta _k=6\cdot 2^{-(2k+4)}$, $k \ge 1$. It is well-known that $\rho ({\mathcal T}_\mathbf {a}|_{V_1 })=\frac{1}{4}$. Thus, after an appropriate normalization of the masks, by Theorem 2, we get that the non-stationary scheme is $C^1$-convergent with the Hölder exponent $\alpha = 2$.

## Further properties

In this subsection, we prove Theorem 1 stated in Sect. 1.2. Its proof is based on the next Proposition 4 that studies the infinite products of certain trigonometric polynomials. The statement of Proposition 4 involves the following concepts.

### Definition 14

A pair of complex numbers $\{z, -z\}$ is called *a pair of symmetric roots* of the algebraic polynomial *q*, if $q(z) = q(-z) = 0$.

Let $\{ q_k, \ k \ge 1\}$ be a sequence of algebraic polynomials of degree *N* and define the function

45 $$\begin{aligned} f(x):= \ \prod _{k=1}^{\infty } p_k(2^{-k}x), \quad p_k(x):=q_k(e^{-2\pi i x}), \quad x \in \mathbb {R}. \end{aligned}$$

By [16], if a sequence of trigonometric polynomials $\{p_k, \ k \ge 1 \}$ is bounded, then this infinite product converges uniformly on each compact subset of $\mathbb {R}$, and hence, *f* is analytic. Possible rates of decay of such functions as $x \rightarrow \infty$ was studied in [60].

### Proposition 4

Assume that the sequence of trigonometric polynomials $\{p_k, \ k \ge 1\}$ with $p_k(0)=1$, $k \ge 1$, converges to a trigonometric polynomial *p* that has no symmetric roots on $\mathbb {R}$. If the function *f* in (45) satisfies $f(x) = o(x^{-\ell })$ for $\ell \ge 0$ and $x \rightarrow +\infty$, then $\delta _{k} = o(2^{-\ell k})$ as $k \rightarrow \infty$, where

$$\begin{aligned} \delta _k=\max _{j=0, \ldots , \ell } 2^{-jk} \frac{|D^j p_k(1/2)|}{j!}, \quad k \ge 1. \end{aligned}$$

### Proof

By assumption $f(x) = o(x^{-\ell })$ for points of the form $x = 2^{k-1} d + t$, where *d* is a fixed natural number, *t* is an arbitrary number from $[0, \sigma ]$, $\sigma >0$, and $k \rightarrow \infty$. Next, we choose these parameters $d \in \mathbb {N}$ and $\sigma > 0$ in a special way.

Firstly, we define $\sigma$. Since $\{p_k, \ k \ge 1\}$ converges to *p*, the sequence $\{p_k, \ k \ge 1\}$ is bounded. Moreover, $p_k(0)=1$, $k \ge 1$, implies that $f(0)=1$. This implies that there are $\sigma \in (0, 1)$ and $C_0 > 0$ such that for every $r \ge 0$ and $R \in \mathbb {N}\cup \{\infty \}$ we have

46 $$\begin{aligned} \left| \, \prod _{j=1}^{R} p_{j+r}\bigl (2^{-j}t\bigr )\, \right| \ \ge \ C_0, \quad t \in [0, \sigma ]. \end{aligned}$$

Next we choose the number *d*. To this end we consider the binary tree defined as follows: the number 1 / 2 is at the root, the numbers 1 / 4 and 3 / 4 are its children, and so on. Every vertex $\alpha$ has two children $\alpha /2$ and $(\alpha +1)/2$. For convenience we shall identify a vertex and the corresponding number. Thus, all vertices of the tree are dyadic points from the interval (0, 1). Indeed, the *n*th level of the tree (i.e., the set of vertices with the distance to the root equal to *n*) consists of points $2^{-n-1}j$, where *j* is an odd number from 1 to $2^{n+1}-1$.

The trigonometric polynomial *p* is 1-periodic and, thus, has at most *N* zeros in [0, 1), and hence, on the tree. Therefore, there is a number *q* such that all roots of *p* on the tree are contained on levels $j \le q$. Since the polynomial *p* has no symmetric roots, at least one of the two children of any vertex of the tree is not a root of *p*. Whence, there is a path of length *q* along the tree starting at the root (all paths are without backtracking) that does not contain any root of *p*. Let $2^{-q-1}d$ be the final vertex of that path, *d* is an odd number, $1\le d \le 2^{q+1}-1$. Denote as usual by $\{x\}$ the fractional part of *x*. Then the sequence $\{2^{-1}d\}, \ldots , \{2^{-q-1}d\}$ does not contain roots of *p*. The sequence $\{2^{-q-2}d\}, \{2^{-q-3}d\}, \ldots$ does not contain them either, because there are no roots of *p* on levels bigger than *q*. Let *n* be the smallest natural number such that $2^{-q-n-1}d < \sigma /2$. We have $p(2^{-1}d)\ldots p(2^{-q-n-1}d) \ne 0$. Since $p_k \rightarrow p$ as $k \rightarrow \infty$, and all $p_k$ are equi-continuous on $\mathbb {R}$, it follows that there is a constant $C_1>0$ such that

47 $$\begin{aligned} \left| \, \prod _{j=1}^{q+n} p_{k+j}\bigl (2^{-j-1}d + 2^{-k-j}x\bigr )\, \right| \ \ge \ C_1, \quad x \in [0, \sigma ], \end{aligned}$$

for sufficiently large *k*. Now we are ready to estimate the value $f(2^{k-1}d + t)$. We have

$$\begin{aligned} \left| \, f\bigl (2^{k-1}d + t\bigr )\, \right|= & {} \left| \, \prod _{j=1}^{k-1} p_{j}\bigl (2^{k-1-j}d + 2^{-j}t\bigr )\, \right| \times \, \left| p_k \bigl ( 2^{-1}d + 2^{-k}t \bigr )\, \right| \\&\times \left| \, \prod _{j=1}^{q+n} p_{k+j}\bigl (2^{-j-1}d + 2^{-k-j}t\bigr )\right| \\&\times \, \left| \, \prod _{j=1}^{\infty } p_{k+q+n+j}\bigl (2^{-j}(2^{-q-n-1}d + 2^{-k-q-n}t)\bigr )\right| . \end{aligned}$$

To estimate the first factor in this product, we note that $2^{k-1-j}d \in \mathbb {Z}$, whenever $j \le k-1$, and hence $p_{j}\bigl (2^{k-1-j}d + 2^{-j}t\bigr ) = p_j(2^{-j}t)$. Thus, the first factor is $\bigl |\, \prod _{j=1}^{k-1} p_{j}(2^{-j}t)\bigr |$, which is, by (46), bigger than or equal to $C_0$, for every $t \in [0, \sigma ]$.

The third factor $\bigl |\, \prod _{j=1}^{q+n} p_{k+j}(2^{-j-1}d + 2^{-k-j}t)\bigr |$, by (47), is at least $C_1$. Finally, the last factor is bigger than or equal to $C_0$. To see this it suffices to use (46) for $R = \infty , r = k+q+n, x = 2^{-q-n}d + 2^{-k-q-n}t$ and note that $x < \sigma$ by the choice of *n*. Thus,

$$\begin{aligned} |f(2^{k-1}d + t)| \ \ge \ C_0^2C_1 \bigl | p_k \bigl ( 2^{-1}d + 2^{-k}t \bigr )\bigr |. \end{aligned}$$

On the other hand, by assumption, $f(2^{k-1}d + t) = o(2^{-\ell k})$ as $k \rightarrow \infty$, consequently $p_k ( 2^{-1}d + 2^{-k}t) = o(2^{-\ell k})$. The number *d* is odd, hence, by periodicity, $p_k ( 2^{-1}d + 2^{-k}t) = p_k ( 1/2 + 2^{-k}t)$. Thus, we arrive at the following asymptotic relation: for every $t \in [0, \sigma ]$ we have

48 $$\begin{aligned} p_k \bigl ( 1/2 + 2^{-k}t\bigr ) \ = \ o\, \bigl ( 2^{-\ell k}\bigr )\quad \text{ as }\ k \rightarrow \infty \, . \end{aligned}$$

This already implies that $D^j p_k(1/2) = o(2^{(j-\ell )k})$ as $k \rightarrow \infty$, for every $j = 0, \ldots , \ell$. Indeed, consider the Tailor expansion of the function $h(t) = p_k \bigl ( 1/2 + 2^{-k}t\bigr )$ at the point 0 with the remainder in Lagrange form:

$$\begin{aligned} h(t) \ = \ \sum _{j=0}^{\ell }\frac{D^j h(0)}{j!}\, t^j\ + \ \frac{D^{\ell +1} h(\theta )}{(\ell +1)!}\, t^{\, \ell +1},\quad t \in [0, \sigma ], \end{aligned}$$

where $\theta = \theta (t) \in [0, t]$. Substituting $D^j h(0) = 2^{-jk} D^j p_k(1/2)$, we get

$$\begin{aligned} p_k(1/2 + 2^{-k}t)= & {} \sum _{j=0}^{\ell }\frac{D^j p_k(1/2)}{j!}\, 2^{-jk}\, t^j\\&+ \ \frac{D^{\ell +1}p_k(1/2 + 2^{-k}\theta )}{(\ell +1)!}\, 2^{-(\ell +1)k}\, t^{\, \ell +1},\quad \ t \in [0, \sigma ] . \end{aligned}$$

First, we estimate the remainder. Since the sequence of trigonometric polynomials $\{p_k, \ k \ge 1\}$ is bounded, the norms $\Vert D^{\ell +1} p_k \Vert _{C[0, \sigma ]}$ do not exceed some constant $C_2$. Therefore,

$$\begin{aligned}&\left| \frac{D^{\ell +1} p_k(1/2 + 2^{-k}\theta )}{(\ell +1)!}\, 2^{-(\ell +1)k}\, t^{\, \ell +1}\right| \\&\quad \le \ \frac{C_2}{(\ell +1)!}2^{-(\ell +1)k}\, \sigma ^{\, \ell +1} \ = \ o(2^{-\ell k})\quad \text{ as } \ k \rightarrow \infty . \end{aligned}$$

Combining this with (48), we get

49 $$\begin{aligned} \left\| \, \sum _{j=0}^{\ell }\frac{D^j p_k(1/2)}{j!}\, 2^{-jk}\, t^j\, \right\| _{\, C([0, \sigma ])}\ = \ o(2^{-\ell k})\quad \text{ as }\quad \ k \rightarrow \infty . \end{aligned}$$

Since, in a finite-dimensional space, all norms are equivalent, the norm of an algebraic polynomial of degree $\ell$ in the space $C([0, \sigma ])$ is equivalent to its largest coefficient. Whence, (49) implies that

50 $$\begin{aligned} \max \limits _{j=0, \ldots , \ell } 2^{-jk} \frac{|D^j p_k(1/2)|}{j!} = o(2^{-\ell k}), \quad k \rightarrow \infty . \end{aligned}$$

$$\square $$

We are finally ready to prove the main result of this section, Theorem 1.

### Proof of Theorem 1

Let $p_k(\omega ):= a_*^{(k)}(e^{-2\pi i \omega })$, $\omega \in \mathbb {R}$, be the symbol of the *k*th mask in the trigonometric form. If the non-stationary scheme converges to a continuous compactly supported refinable function $\phi$, then its Fourier transform $\widehat{\phi }(\omega ) = \int _{\mathbb {R}}\phi (x)e^{-2\pi i x \omega }d x$ is given by

51 $$\begin{aligned} \widehat{\phi }(\omega ) = \prod _{k=1}^{\infty } p_k(2^{-k}\omega ), \quad \omega \in \mathbb {R}. \end{aligned}$$

If $\phi \in C^\ell (\mathbb {R})$, then $\widehat{\phi }(\omega ) = o(\omega ^{-\ell })$ as $\omega \rightarrow \infty$. Since the refinable function of the limit mask $\mathbf {a}$ is stable, it follows that its symbol $a_*(z)$ has no symmetric roots on the unit circle. The claim follows by Proposition 4. Indeed, by definition of $p_k$, we get by (50)

$$\begin{aligned} \max \limits _{j=0, \ldots , \ell } 2^{-jk} |D^j a_*^{(k)}(-1)| = o(2^{-\ell k}) \quad \hbox {as} \quad k \rightarrow \infty , \end{aligned}$$

which completes the proof.$\square$
